# High Crystallinity 2D π–d Conjugated Conductive Metal–Organic Framework for Boosting Polysulfide Conversion in Lithium–Sulfur Batteries

**DOI:** 10.1002/advs.202302518

**Published:** 2023-07-28

**Authors:** Tong Guo, Yichen Ding, Chang Xu, Wuxin Bai, Shencheng Pan, Mingliang Liu, Min Bi, Jingwen Sun, Xiaoping Ouyang, Xin Wang, Yongsheng Fu, Junwu Zhu

**Affiliations:** ^1^ Key Laboratory for Soft Chemistry and Functional Materials of Ministry of Education Nanjing University of Science and Technology Nanjing 210094 P. R. China; ^2^ Key Laboratory of Low Dimensional Materials and Application Technology School of Materials Science and Engineering Xiangtan University Xiangtan 411105 P. R. China

**Keywords:** conductive metal–organic frameworks, functionalized separator, lithium–sulfur batteries, polysulfides

## Abstract

The catalytic performance of metal–organic frameworks (MOFs) in Li‐S batteries is significantly hindered by unsuitable pore size, low conductivity, and large steric contact hindrance between the catalytic site and lithium polysulfide (LPSs). Herein, the smallest π‐conjugated hexaaminobenzene (HAB) as linker and Ni(II) ions as skeletal node are in situ assembled into high crystallinity Ni‐HAB 2D conductive MOFs with dense Ni‐N_4_ units via dsp^2^ hybridization on the surface of carbon nanotube (CNT), fabricating Ni‐HAB@CNT as separator modified layer in Li‐S batteries. As‐obtained unique π‐d conjugated Ni‐HAB nanostructure features ordered micropores with suitable pore size (≈8 Å) induced by HAB ligands, which can cooperate with dense Ni‐N_4_ chemisorption sites to effectively suppress the shuttle effect. Meanwhile, the conversion kinetics of LPSs is significantly accelerated owing to the small steric contact hindrance and increased delocalized electron density endued by the planar tetracoordinate structure. Consequently, the Li‐S battery with Ni‐HAB@CNT modified separator achieves an areal capacity of 6.29 mAh cm^−2^ at high sulfur loading of 6.5 mg cm^−2^ under electrolyte/sulfur ratio of 5 µL mg^−1^. Moreover, Li‐S single‐electrode pouch cells with modified separators deliver a high reversible capacity of 791 mAh g^−1^ after 50 cycles at 0.1 C with electrolyte/sulfur ratio of 6 µL mg^−1^.

## Graphical abstract

High crystallinity π–d conjugated hexaaminobenzene‐based 2D conductive MOF (Ni‐HAB) is in situ assembled onto the surface of carbon nanotube (Ni‐HAB@CNT) as separator modified layer in lithium–sulfur batteries. Ni‐HAB with high conductivity, ordered micropores (≈8 Å), and abundant Ni‐N active sites can effectively inhibit the shuttle effect of polysulfides and accelerate their reaction kinetics.

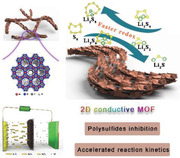

## 1 Introduction

The proposal of carbon peaking and carbon neutrality has attracted more and more attention to developing new energy storage technologies. Lithium–sulfur batteries (LSBs) are the most promising next‐generation energy storage batteries due to their high theoretical energy density (2600 Wh kg^−1^) and natural abundance of sulfur.^[^ ^1^, ^2^ ^]^ Nevertheless, the commercialization of Li‐S batteries is still hindered by unsatisfactory energy density and poor cycling stability of the device due to their intrinsic defects, such as i) the low utilization of active material caused by the insulation characteristic of S (5 × 10^−30^ S cm^−1^) and Li_2_S (10^−13^ S cm^−1^),^[^ ^3^ ^]^ ii) the volume strain of electrode structure due to the volume change of S (2.07 g cm^−3^) and Li_2_S (1.66 g cm^−3^),^[^ ^4^ ^]^ and iii) the notorious shuttle effect because of the high solubility of lithium polysulfides (LPSs) in weakly polar ether solvents.^[^ ^5^ ^]^ Consequently, Li‐S batteries suffer from sluggish reaction kinetics and poor cycle life.

Significant efforts have been devoted to solving the above issues to improve the electrochemical performance of LSBs, including using heteroatom‐doped porous carbon materials,^[^ ^6^ ^]^ covalent–organic frameworks (COFs),^[^ ^7^ ^]^ transition metal compounds,^[^ ^8^, ^9^, ^10^ ^]^ MXene materials,^[^ ^11^, ^12^ ^]^ and metal–organic frameworks (MOFs) as encapsulant for sulfur, constructing polypropylene (PP) separator modification layer with both adsorption and catalytic activity for LPSs,^[^ ^13^ ^]^ designing multifunctional electrolyte additives,^[^ ^14^ ^]^ and so on.^[^ ^15^ ^]^ Among them, the rational design of modified layer on the PP separator is a simple and effective way to improve the utilization of S and suppress the shuttle effect of LPSs. In principle, an ideal modified layer should meet the following requirements: 1) high permselectivity which can allow only lithium ions to freely migrate, while LPSs are not allowed; 2) good electrical conductivity that can ensure high electrochemical activity of LPSs; and 3) abundance of exposed electrocatalytic activate sites that can improve the reaction kinetics of LPSs. To date, more than a hundred materials have been fabricated as PP modified layers through a variety of ways to alleviate a series of problems of LSBs.^[^ ^4^ ^]^ MOFs with high hierarchical porosity, tunable pore parameters, rich polar functional groups, and abundant metal reaction sites have shown great potential in the field of electrochemical energy conversion and storage.^[^ ^7^, ^13^, ^16^ ^]^ ZIF‐67,^[^ ^17^ ^]^ ZIF‐8@CNT,^[^ ^18^ ^]^ copper‐based MOF@PVDF,^[^ ^19^ ^]^ Zr‐Fc MOF,^[^ ^20^ ^]^ and Ce‐MOF/CNT^[^ ^21^ ^]^ have been applied successfully to PP separator modified layer and have been proven effective in suppressing shuttle effect and improving battery performance. However, the central transition metal atoms (M) of most MOFs are situated in a tetrahedral or octahedral coordination environment, increasing the steric contact hindrance between the catalytic site and LPSs, consequently limiting the catalytic activity. Moreover, the lower dz^2^(M)–π(Ligand) orbital overlap disrupts electron transport within the molecule, resulting in terrible electrical conductivity. The lack of electrical conductivity and large steric effects in vast majority of MOFs greatly limits the electrochemical activity of built‐in metal atoms, leading to the low utilization of catalysts in LSBs.

2D conductive MOFs (c‐MOFs) formed by the self‐assembly of metal centers and π‐conjugated ligands possess excellent electrical conductivity induced by the π–d conjugation effect, which is expected to be an ideal separator modified layer.^[^ ^22^, ^23^ ^]^ However, so far relatively little attention has been paid to the application of c‐MOFs in LSBs. Meanwhile, the previously reported 2D c‐MOFs mainly focus on large molecular ligands such as 2,3,6,7,10,11‐hexaiminotriphenylene,^[^ ^24^ ^]^ which results in the formation of big sized holes and a low density of catalytic sites, thereby limiting their capability to effectively capture and catalyze LPSs. Therefore, using a relatively small π‐conjugated hexaaminobenzene (HAB) linkers can not only construct smaller crystal internal pores to enhance the trapping ability for LPSs, but also further result in a framework with a high density of active sites.

Herein, for the first time, the smallest π‐conjugated ligand HAB as organic connection and Ni(II) ions as skeletal node are in situ constructed into high crystallinity Ni‐HAB 2D c‐MOFs on the surface of carbon nanotube (CNT), fabricating Ni‐HAB@CNT. The high crystallinity Ni‐HAB 2D c‐MOFs feature dense arrangement dsp^2^‐hybridized Ni‐N_4_ units, uniform sub‐nanometer pore size, lower steric contact hindrance caused by square‐planar coordination geometry, and excellent conductivity due to large conjugation system. Combining theoretical calculation analysis and in situ characterization (in situ Raman and in situ XRD), the Ni‐HAB can not only increase the adsorption of LPSs, but also accelerate the formation and the decomposition of Li_2_S during sulfur reduction reaction (SRR) and sulfur evolution reaction (SER), respectively. Consequently, the LSBs equipped with Ni‐HAB@CNT separators show extraordinary long‐term cyclability with capacity retention of 85.2% after 200 cycles at 0.2 C and the high area capacity of 6.29 mAh cm^−2^ with a sulfur areal loading of 6.5 mg cm^−2^ under electrolyte/sulfur (E/S) ratio of 5 µL mg^−1^. Moreover, the pouch cell delivers a quite stable and high reversible capacity of 791 mAh g^−1^ after 50 cycles at 0.1 C, even with the low electrolyte usage (E/S = 6 µL mg^−1^).

## 2 Results and Discussion

**Figure 1:**
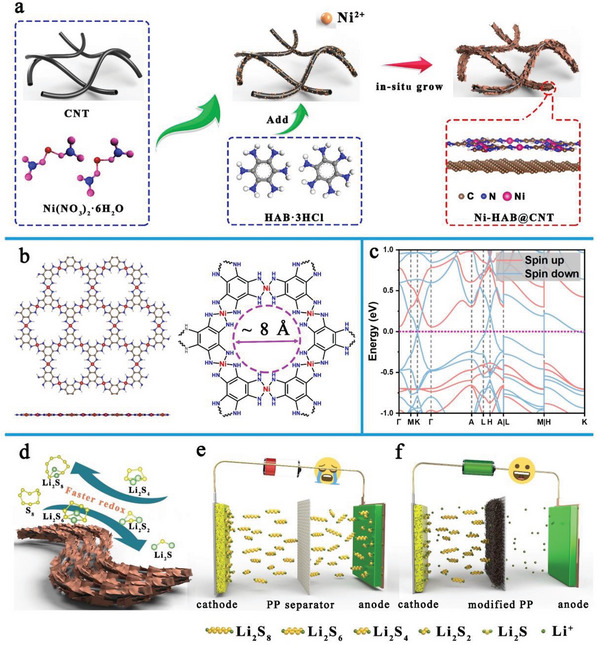
a) Schematic diagrams of the synthesis of Ni‐HAB@CNT; b) top and side views of Ni‐HAB; c) calculated electronic band structure of bulk Ni‐HAB along the high symmetry Γ‐M‐K‐Γ‐A‐L‐H‐A|L‐M|H‐K points. The Fermi energy in the band structure is as zero; d) schematic illustrations of sulfur transformation process on Ni‐HAB@CNT; e) traditional PP separator experiences serious shuttling effect; f) Ni‐HAB@CNT modified separator greatly inhibits LPSs shuttling.

The synthetic process of Ni‐HAB@CNT is schematically illustrated in **Figure** **1**a. HAB was used as the organic ligand, which was fabricated by stepwise amination of 4‐nitroaniline according to our previous report.^[^ ^25^ ^]^ The few‐layered Ni‐HAB c‐MOF nanosheets were in situ grown on the surface of acid‐treated CNT through facile adsorption followed by a solvothermal process (see the Experimental Section for more details). Prior to the growth of Ni‐HAB c‐MOF, the commercial CNTs were first treated by acid reflux to attach oxygen‐containing groups (such as carboxyl and hydroxyl) and introduce defects (Figure S1d, Supporting Information), facilitating the surface nucleation and anchoring for the Ni‐HAB c‐MOF. As shown in Figure 1b, The Ni‐HAB c‐MOF is formed by the periodic connection of square planar dsp^2^‐hybridized coordination of Ni(II) and π‐conjugated HAB ligands, exhibiting a 2D honeycomb layered structure and uniform pore distribution. The pore size is about 8 Å, smaller than the diameter of the LPSs solvated structure and larger than the diameter of the Li‐ion solvated structure.^[^ ^23^, ^26^ ^]^ Therefore, Ni‐HAB 2D MOFs have an inherent advantage in blocking liquid‐phase LPSs diffusion. In addition, the electronic band structure of bulk Ni‐HAB (*a* = *b* = 13.34 Å; *c* = 3.33 Å; *α* = *β* = 90.00°; *γ* = 120.00°) obtained from the DFT calculations within the HSE06 (AEXX = 0.25) functional and corresponding k‐points path along the high symmetry points in the first Brillouin zone is displayed in Figure 1c and Figure S2 (Supporting Information). The valence bands cross the Fermi level, indicating the nature metallic character of Ni‐HAB. The metallic character can be ascribed to the increased delocalized electron density induced by the maximizing dz^2^(M)–π(Ligand) orbital overlap.^[^ ^27^, ^28^, ^29^ ^]^ In addition, the conductivity of 90 ± 15 S m^−1^ of Ni‐HAB pellet was also observed in the experiment, which is consistent with the previous literature reports.^[^ ^23^, ^27^ ^]^ Furthermore, the unique dsp^2^ hybridization endues Ni(II) with planar tetracoordinate structure, which can minimize the steric contact hindrance between the catalytic site and LPSs, further enhancing the catalytic activity. The introduction of CNT substrate can not only effectively suppress the random agglomeration of Ni‐HAB nanoparticles triggered by nanoeffects, exposing more high active centers, but also allow the composite to inherit the inborn weaving advantage of CNT and achieve uniform coverage of the separator surface. Combining the intrinsic metallic properties, abundant Ni‐N sites, and appropriate pore size of Ni‐HAB, along with the advantages of easy integration into a net structure and ultra‐high conductivity of the CNT substrate, it is expected that the utilization of Ni‐HAB@CNT as the modification layer for the PP separator will significantly enhance the redox reactivity of LPSs and effectively suppress the shuttle effect (Figure 1d–f).

**Figure 2:**
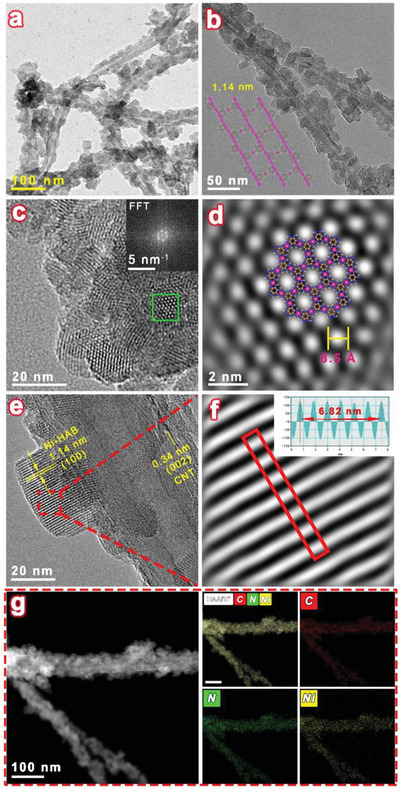
a,b) TEM images of Ni‐HAB@CNT; c) HRTEM image of Ni‐HAB. Inset: Fast Fourier transform (FFT) image; d) IFFT image of selected area in (c); e) HRTEM images of Ni‐HAB@CNT; f) IFFT of selected area and lattice spacing profiles at selected area of (e); g) HAADF‐STEM image and corresponding elemental mappings of overlap, carbon, nitrogen, and nickel in Ni‐HAB@CNT.

The morphology and structural characteristics of the as‐prepared Ni‐HAB@CNT were characterized by scanning electron microscopy (SEM) and transmission electron microscopy (TEM). The acid‐treated CNTs possess a smooth surface and a diameter distribution between 20 and 40 nm (Figure S1a,b, Supporting Information). The (002) crystalline plane of carbon and defect sites are also clearly displayed (Figure S1c,d, Supporting Information). As shown in **Figure** **2**a,b and Figure S3 (Supporting Information), for the Ni‐HAB@CNT, Ni‐HAB c‐MOF nanosheets with the mean size of 30 nm are tightly attached to the surface of CNTs even after high‐power ultrasonication owing to chemical interaction between the nickel metal centers and functionalized CNTs. Noteworthy, the Ni‐HAB nanosheets adhered to the CNTs have excellent crystallinity, and the layer spacing belonging to the family of crystallographic planes can be clearly identified at low magnification (Figure 2b). The high‐resolution TEM image and inverse fast Fourier transformation (IFFT) image further display the lattice fringe with *d*‐spacing of 1.14 and 0.34 nm, corresponding to the (001) plane of Ni‐HAB and (002) plane of CNT, respectively (Figure 2e,f and Figure S4, Supporting Information). Furthermore, the corresponding high‐angle annular dark‐field scanning‐transmission electron microscopy (HAADF‐STEM) image and elemental mappings confirm the uniform distribution of three elements of C, N, and Ni on Ni‐HAB@CNT (Figure 2g). Meanwhile, pure Ni‐HAB c‐MOF has been prepared in the same condition. The HRTEM images of Ni‐HAB (Figure 2c,d) clearly show hexagonal pores of ≈8.5 Å, corresponding well with the eclipsed model (Figure S4, Supporting Information). However, the pure Ni‐HAB nanosheets with excellent crystallinity are severely agglomerated due to strong π–π stacking interactions between layers (Figure S5, Supporting Information).

**Figure 3:**
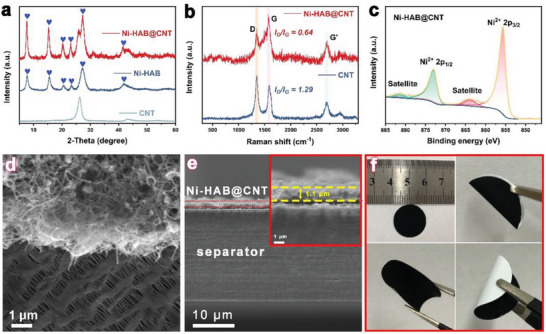
a) XRD patterns of CNT, Ni‐HAB, and Ni‐HAB@CNT; b) Raman spectra of CNT and Ni‐HAB@CNT; c) high‐resolution XPS spectrum of Ni 2p of Ni‐HAB@CNT; d,e) cross‐section SEM images of Ni‐HAB@CNT separator; f) photos showing the as‐prepared Ni‐HAB@CNT modified separator and the flexibility.

Powder X‐ray diffraction (PXRD) and Raman spectroscopy were applied to verify the crystallographic structure and chemical compositions. The XRD pattern of Ni‐HAB@CNT is in good agreement with the pure Ni‐HAB c‐MOF and CNT, indicative of significant crystallinity (**Figure** **3**a). The Raman spectra (Figure 3b) of Ni‐HAB@CNT consist of typical D (1350 cm^−1^) and G (1582 cm^−1^) peaks with an overtone of G mode, namely, 2D (2629 cm^−1^). Noteworthy, Ni‐HAB@CNT displays a lower *I*_d_/*I*_g_ intensity ratio (0.64) than that of CNT (1.29), which can be attributed to the fact that the heterogeneous structure exposes more benzene‐ring‐related vibrations. Consistent with the XRD results, the Raman spectra of Ni‐HAB@CNT exhibit the same peak positions as those of the pure Ni‐HAB c‐MOF (Figure S6, Supporting Information). The structural confirmation was also analyzed using X‐ray photoelectron spectroscopy (XPS). The survey spectra of XPS for Ni‐HAB@CNT and Ni‐HAB reveal the presence of Ni, C, N, and O elements (Figure S7, Supporting Information). The O element in Ni‐HAB may come from adsorbed water or oxygen. As shown in Figure 3c, high‐resolution Ni 2p spectrum of Ni‐HAB@CNT shows the Ni (II) oxidation state.^[^ ^27^, ^30^ ^]^ As shown in Figure S8 (Supporting Information), the pristine PP separator exhibits large pores ranging from several tens to hundreds of nanometers. In sharp contrast, there is a dense microporous layer of Ni‐HAB@CNT tightly covered on the surface of PP separator for the Ni‐HAB@CNT/PP (Figure 3d). Meanwhile, the EDS mappings (Figure S9, Supporting Information) confirm the homogeneous distributions of Ni, C, and N elements on the separator. It is well known that the volume and the intrinsic liquid absorption capacity of the modification layer directly affect the energy density of the cell, thus nitrogen adsorption–desorption isotherms were used to identify surface area and pore structure. As shown in Figure S10 and Table S1 (Supporting Information), Both the Brunauer–Emmett–Teller (BET) specific surface area and pore volume of Ni‐HAB@CNT are between that of CNT and Ni‐HAB. Compared with Ni‐HAB and CNT, Ni‐HAB@CNT exposes more holes smaller than 3 nm, which could effectively block the diffusion of polysulfide. In addition, the typical cross‐sectional SEM image further reveals that the thickness of Ni‐HAB@CNT membrane is only 1.1 µm (inset image of Figure 3e). Such a low pore volume and thin thickness can effectively reduce the damage to the battery energy density caused by the introduction of the modification layer. Furthermore, Ni‐HAB@CNT modified PP separator exhibits good flexibility benefiting from the intimate interactions between the Ni‐HAB@CNT layer and PP membrane, ensuring the cycling stability of LSBs (Figure 3f). An excellent separator modification layer should block the diffusion of polysulfides without hindering the diffusion of lithium ions to meet the needs of high‐rate charge and discharge. The electrical impedance spectroscopy measurements at different temperatures indicate that the Ni‐HAB@CNT modified layer has no impact on the transport of Li^+^ (Figure S11, Supporting Information).

Ni‐HAB@CNT has abundant Co‐N active sites, which can endow Ni‐HAB@CNT modified layer with strong chemical adsorption capacity for LPSs, inhibiting the shuttle of LPSs and enhancing stability of LSBs. To clearly show effective suppression of Ni‐HAB@CNT on shuttle effect of LPSs, the visual permeation experiments were conducted in an H‐typed glass apparatus with one side filled with ethers solvent and the other side filled with 0.05 m Li_2_S_6_ solution, which was separated by Ni‐HAB@CNT/PP and PP membranes, respectively (Figure S12, Supporting Information). Apparently, the routine PP separator fails to prevent the shuttle of Li_2_S_6_. It can be clearly seen that the permeation of LPSs occurs within 3 h and notably increases with the time delay. In sharp contrast, for the Ni‐HAB@CNT modified PP separator, there is no permeation of Li_2_S_6_ even after 12 h, indicating that Ni‐HAB@CNT barrier can effectively suppress the shuttle effect of LPSs. Meanwhile, when Ni‐HAB@CNT was added to the Li_2_S_6_ solution and left for 6 h, the color of the Li_2_S_6_ solution changed from bright yellow to nearly transparent (inset of Figure S13, Supporting Information). The UV–vis spectra (Figure S13, Supporting Information) confirmed that the Li_2_S_6_ in the solution was almost completely adsorbed by Ni‐HAB@CNT. In contrast, for CNT and Ni‐HAB, the Li_2_S_6_ solutions remained pale yellow after undergoing a similar process, and the characteristic peak of Li_2_S_6_ was distinct in UV–vis spectra. These results demonstrate the strong chemical adsorption between Li_2_S_6_ and Ni‐HAB@CNT.

**Figure 4:**
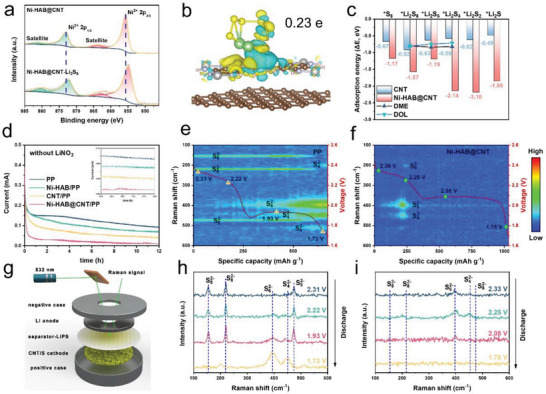
a) Ni 2p XPS spectra of Ni‐HAB@CNT before and after adsorption of Li_2_S_6_; b) density functional theory (DFT) simulations: the optimized adsorption configurations of Li_2_S_6_ interacted with Ni‐HAB@CNT and corresponding different charge density (DCD) analysis, the value of the isosurface is set to be 0.001 e Å^−3^; c) comparison of adsorption energies of Li_2_S*_n_* with Ni‐HAB@CNT, CNT, and electrolyte solvent molecules (DME and DOL); d) the shuttle currents of Li‐S batteries with PP, Ni‐HAB/PP, CNT/PP, and Ni‐HAB@CNT/PP as the separators; e,f) in situ time‐resolved Raman spectra obtained during the discharging processes with PP and Ni‐HAB@CNT modified separators. g) Schematic illustration of a Li‐S battery toward in situ Raman tests; h,i) selected Raman spectra of Li‐S cells based on PP and Ni‐HAB@CNT modified separators. The red curves represent the discharging processes.

XPS measurements were performed to further evaluate the strong chemical interactions between Ni─N bonds and LPSs. **Figure** **4**a and Figure S14 (Supporting Information) exhibit the high‐resolution Ni 2p XPS spectra of Ni‐HAB@CNT and Ni‐HAB before and after the Li_2_S_6_ adsorption test, respectively (Ni‐HAB@CNT‐Li_2_S_6_, Ni‐HAB‐Li_2_S_6_). After being interacted with Li_2_S_6_, the Ni 2p peaks significantly shift to lower binding energy, which denotes the electron transfer from S to the Ni (II) to form strong chemical interaction between Li_2_S_6_ and Ni‐N sites. In addition, the N 1s peaks shifted towards a lower binding energy range after the formation of a composite with Li_2_S_6_, indicating an increase in electron cloud density around the nitrogen atom due to the formation of the Li─N bond (as shown in Figure S15, Supporting Information). Using DFT calculations to in‐depth analysis of electron transfer between Ni‐HAB@CNT and Li_2_S_6_ at the atomic level, Figure 4b shows the different charge density (DCD) distribution, where charge depletion and accumulation are depicted by cyan and yellow, respectively. It can be seen that the electron density near Ni (II) increases and near S decreases, indicating the strong Lewis acid–base interaction between each other (Figure S16b, Supporting Information). Corresponding Bader charge analysis further clearly shows that Ni‐HAB@CNT gains about 0.23 electrons from S (Figure 4b). In contrast, a negligible amount of charge transfer between Li_2_S_6_ molecule and CNT (Figures S16a and S17, Supporting Information).

Next, the binding energies between Ni‐HAB@CNT and different S‐related molecules including S_8_ and Li_2_S*_n_* (*n* = 1, 2, 4, 6, 8) are investigated, and these results are compared with the similar reactions on CNT as reference. The optimal adsorption configuration corresponding to the adsorption energy is shown in Figures S18 and S19 (Supporting Information). As shown in Figure 4c, the adsorption energies of intermediate LPSs and S_8_ on Ni‐HAB@CNT are higher than those of CNT, suggesting that coupling Ni‐HAB with the CNT substantially enhanced anchoring ability for S‐related species, mainly through Ni−N bonds. Given the solubility of long‐chain LPSs in electrolytes, we assess the binding affinity between LPSs and electrolyte solvent molecules, namely 1,2‐dimethoxyethane (DME) and 1,3‐dioxolane (DOL). Clearly, the binding energies of Ni‐HAB@CNT to the long‐chain LPSs are higher than those of electrolyte solvents to the LPSs. The shuttle current of LSBs with diverse separators was recorded to further estimate the ability of LPSs inhibition. The concentration of LPSs reaches a maximum at a potential of 2.38 V during the charge/discharge process, resulting in a maximum shuttle current.^[^ ^14^, ^31^ ^]^ The shuttle current of the cell with Ni‐HAB@CNT modified separator is almost negligible (9 µA), which is far less than that of the other three cells (Ni‐HAB, CNT, and PP, Figure 4d). It is clear that the cell with Ni‐HAB@CNT modified separator has excellent trapping capacity for LPSs, significantly inhibiting the diffusion of LPSs to the anode electrode and leading to improved electrochemical performance.

To deeply probe the role of Ni‐HAB@CNT in blocking the shuttle of polysulfides, in situ Raman spectroscopy was conducted to detect the polysulfides shuttling to the anode side in real‐time. The configuration of Raman test device is illustrated in Figure 4g. Generally, Li_2_S*_n_* (4 < *n* < 6) can be clearly distinguished with Raman spectroscopy according to the typical two‐plateau voltage profile. As the contour map is shown in Figure 4e, for the discharge process of the cell with PP separator, the signals of S_8_^2−^ (peaks located at 153, 220, and 471 cm^−1^) are detected in the earliest stages (>2.33 V), indicating that long‐chain LPSs produced by CNT/S cathode are easily formed and shuttled, which is in agreement with previous studies.^[^ ^32^, ^33^ ^]^ During the further continuous discharging, the strong characteristic peaks at around 400, 512, and 450 cm^−1^ appear and remain until the end of the discharge (1.93 → 1.7 V), which are representative of mid‐length and short‐chain LPSs like Li_2_S_6_ and Li_2_S_4_ (Figure 4h).^[^ ^34^ ^]^ It should be noted that S_8_^2−^ signals are almost throughout the whole discharge process and the peak intensities of S_6_^2−^ and S_4_^2−^ reach a maximum at the end of the discharge process, indicative of a severe LPSs shuttle behavior. In contrast, the cell with Ni‐HAB@CNT modified separator only shows small signals of LPSs during the entire discharge process (Figure 4f, i). Furthermore, in the control cell (PP, Figure 4e), the lower discharge plateau is significantly shorter and the discharge voltage is lower compared to the cell modified with Ni‐HAB@CNT (Figure 4f), indicating inferior redox accessibility and relatively sluggish reaction kinetics in the control cell under lean electrolyte. These consequences strongly demonstrate that Ni‐HAB@CNT modified layer can effectively suppress the shuttle effect of LPSs through the synergistic effect of abundant chemisorption sites and dense physical barriers.

**Figure 5:**
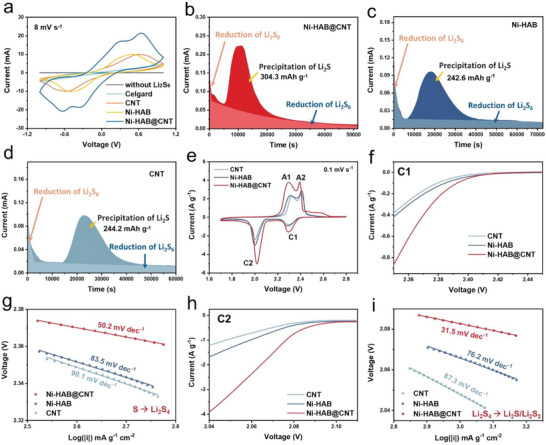
a) CV curves of Li_2_S_6_ symmetric cells with different electrodes; b–d) potentiostatic discharge plots of different cells at 2.05 V; e) CV profiles of Li‐S cells with different separators at a scan rate of 0.1 mV s^−1^; f) the enlarged cathodic peak in the range of 2.35–2.45 V and g) corresponding Tafel profiles; h) the enlarged cathodic peak in the range of 2.04–2.11 V and i) corresponding Tafel profiles.

To verify the electrocatalytic properties of Ni‐HAB@CNT composite toward the polysulfide conversion, the cyclic voltammetry (CV) test of Li_2_S_6_‐Li_2_S_6_ symmetric cells was conducted in the voltage range of −1.0 to 1.0 V. As shown in **Figure** **5**a, the symmetrical cell with Ni‐HAB@CNT modified separator features four distinct peaks with a higher redox current and smaller polarization than those of cells with Ni‐HAB, CNT and bare PP separator at a scan rate of 8 mV s^−1^, demonstrating accelerated LPSs redox kinetics.^[^ ^32^, ^35^ ^]^ Importantly, the CV profiles of the fifth cycle overlapped with the initial cycle, indicative of an outstanding catalytic activity and stability of Ni‐HAB@CNT toward LPSs redox (Figure S20a, Supporting Information).^[^ ^36^ ^]^ The catalytic capability of Ni‐HAB@CNT toward LPSs conversion was further proved by CV test under different scan rates (Figure S20b, Supporting Information). It can be seen that the current density of the CV curves enhances with the increase of scan rate, implying a diffusion‐controlled redox behavior. Impressively, the CV curve still displays four clear redox peaks, further demonstrating the fast LPSs redox kinetics.^[^ ^37^ ^]^

To further investigate the role of Ni‐HAB@CNT in the liquid‐solid conversion of LPSs, Li_2_S nucleation and growth test were also carried out. The potentiostatic discharging curves of Li_2_S precipitation at 2.05 V based on different electrode materials (carbon paper matrix coated by CNT, Ni‐HAB, or Ni‐HAB@CNT) are presented in Figure 5b–d and the capacity was calculated based on the mass of S in electrolyte. Obviously, the responsivity of Li_2_S nucleation for the cell with Ni‐HAB@CNT modified separator is earlier than that of the cell with Ni‐HAB and CNT modified separators, suggestive of a relatively fast kinetics of the liquid‐solid conversion.^[^ ^38^, ^39^ ^]^ Moreover, the nucleation capacity (Li_2_S_4_ → Li_2_S) on Ni‐HAB@CNT (304.3 mAh g^−1^) is much larger than those on Ni‐HAB (242.6 mAh g^−1^) and CNT (244.2 mAh g^−1^) even at a shorter nucleation and growth time, further revealing that the effective deposition of Li_2_S is induced by Ni‐HAB@CNT catalyst.^[^ ^37^ ^]^ Assessing the rate of Li_2_S dissolution on the catalytic matrix is another crucial indicator for evaluating the kinetic aspects of LPS conversion. As shown in Figure S21 (Supporting Information), during the potentiostatic charge measurement, Ni‐HAB@CNT exhibits a significantly higher dissolution current response, earlier dissolution time, and larger dissolution capacity compared to both Ni‐HAB and CNT.

Figure 5e presents the CV profiles of the Li‐S coin cells with different separators in the voltage window of 1.7–2.8 V under a scan rate of 0.1 mV s^−1^. There are two typical reduction peaks at C1 (≈2.3 V) and C2 (≈2.0 V) corresponding to the formation of soluble LPSs intermediates and solid Li_2_S_2_/Li_2_S, respectively. While the two obvious partially overlapped oxidation peaks A1 (≈2.3 V) and A2 (≈2.4 V) ascribed to the reversible formation of Li_2_S*_n_* and S_8_.^[^ ^40^, ^41^ ^]^ Note that the cell with Ni‐HAB@CNT modified separator exhibits the largest response peak current and the lowest reaction polarization for all redox peaks (Figure 5e and Figure s22, Supporting Information), indicating a significantly reinforced sulfur redox kinetics. This may be ascribed to the synergistic effect between CNT and Ni‐HAB, where the introduction of CNTs enhances the composite electrical conductivity by reducing the density of localized electrons in the materials, while the abundant Ni‐N active centers in Ni‐HAB can improve the bidirectional conversion of polysulfides.

For quantitative analysis, Tafel plots of the corresponding redox peaks were obtained.^[^ ^42^ ^]^ For the peak C1 (S_8_ → Li_2_S_4_), the Tafel slopes for Ni‐HAB@CNT, Ni‐HAB, and CNT are 50.2, 183.5, and 90.1 mV dec^−1^, respectively (Figure 5f,g). For the peak C2 (Li_2_S_4_ → Li_2_S_2_/Li_2_S), they are 31.5, 76.2, and 81.3 mV dec^−1^, respectively (Figure 5h,i). The smallest Tafel slope for the cell with Ni‐HAB@CNT modified separator indicates more facile lithiation process of sulfur. Meanwhile, the Tafel curves in Figure s23 (Supporting Information), supporting information also verify that the Ni‐N sites in Ni‐HAB c‐MOFs can considerably accelerate the oxidation kinetics for the reaction of Li_2_S/Li_2_S_2_ → S. It is worth mentioning that the onset potential for Ni‐HAB@CNT‐mediated polysulfide redox is advanced in both discharge and charge processes, demonstrating the high catalytic activity of Ni‐HAB@CNT as well.

**Figure 6:**
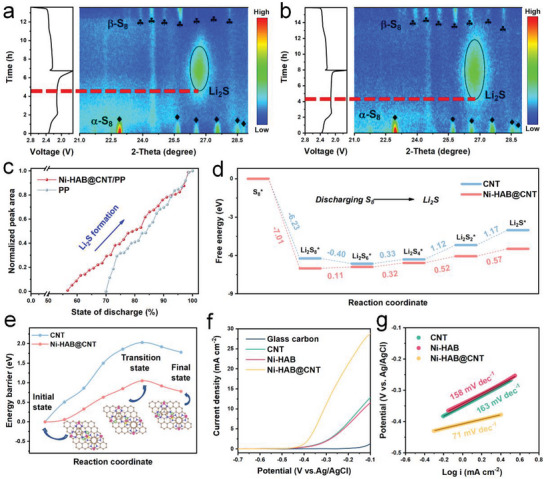
In situ XRD characterization in contour plots of the Li‐S cells and corresponding charge–discharge profiles (left) a) without and b) with Ni‐HAB@CNT modified layer during the initial cycle at the current rate of 0.1 C; c) the peak area quantified evolution of crystalline Li_2_S phase as a function of discharge states; d) Gibbs free energy profiles for the discharging process on Ni‐HAB@CNT and CNT; e) decomposition energy barriers for Li_2_S → LiS + Li on Ni‐HAB@CNT and CNT for different adsorbate configurations (insets: the initial, transition, and final state structures); f) LSV curves and g) the corresponding Tafel plots of Li_2_S oxidation with different electrodes at 5 mV s^−1^.

In order to dig deeper into the detailed reaction kinetics and mechanism of the reaction process, in situ XRD measurements were performed to directly monitor the sulfur species evolution based on Li‐S cells with different separators. **Figure** **6**a depicts the contour XRD patterns as a function of state of the first discharge/charge. Initially, the diffraction peaks at 22.91°, 25.70°, 26.56°, 27.56°, 28.52°, and 28.80° are attributed to the elemental orthorhombic *α*‐S_8_ (JCPDS No. 00‐008‐0247).^[^ ^43^, ^44^ ^]^ Once the lithiation process starts, the intensity of the S_8_ peaks gradually becomes weak toward the end of the upper‐discharge plateau (2.4–2.2 V), accompanied by the formation of soluble LPSs. Subsequently, the obvious diffraction peak at around 26.7° of cubic Li_2_S phase (JCPDS 023‐0369) emerges with increasing intensity.^[^ ^45^ ^]^ The amount of Li_2_S gradually increases as the lithiation proceeds and reaches the maximum at the end of discharge (1.7 V). Reversely, during the delithiation process, Li_2_S is gradually oxidized to S_8_, accompanied by the appearance of diffraction peaks of peaks (with plum‐shaped symbols) representing monoclinic *β*‐S_8_ (JCPDS No. 01‐071‐0137).^[^ ^46^, ^47^ ^]^

The above results are in good agreement with the work mechanism of LSBs in liquid‐phase electrolytes. Noteworthy, although we observe the formation of cubic Li_2_S phase and reformation of sulfur during the discharge and charge (Figure 6a,b, respectively), the evolution rate of S_8_/Li_2_S is quite different for PP and Ni‐HAB@CNT/PP systems. First, the cell with Ni‐HAB@CNT modified PP separator possesses higher capacities and smaller polarization compared to the cell with bare PP separator. Second, integrating the major peak area of Li_2_S by normalization as a function of discharge state is shown in Figure 6c. It is worth noting that the emergence of Li_2_S for the cell with Ni‐HAB@CNT modified PP separator occurs earlier than that of the cell with bare PP, indicating that the Ni‐HAB@CNT can significantly improve the conversion kinetics of LPSs. Besides, the cell with Ni‐HAB@CNT modified PP separator can accelerate the nucleation and growth of Li_2_S, resulting in higher strength and larger peak areas of Li_2_S, which is consistent with the previous Li_2_S deposition experimental results. Moreover, the intensities of *β*‐S_8_ peaks for the cell are significantly reduced compared to those observed at the beginning of the discharge (*α*‐S_8_), which may be attributed to the decreased size of sulfur particles.^[^ ^44^ ^]^ However, the cell with Ni‐HAB@CNT/PP exhibited higher intensities of *β*‐S_8_ peaks than that of PP, which further demonstrates the effective suppression of shuttle effect and the enhanced oxidation reaction process of Li_2_S achieved by Ni‐HAB@CNT.

DFT calculations were conducted to comprehensively explore redox kinetics of the sulfur species.^[^ ^7^ ^]^ The S reduction pathways on both CNT and Ni‐HAB@CNT substrates were investigated. Figure 6d shows the relative Gibbs free energy landscape for the evolution profiles from S_8_ stepwise lithiation to Li_2_S. The corresponding optimized structures of the intermediates are shown in Figures S18 and S19 (Supporting Information). It is obvious that the first step from S_8_ to Li_2_S_8_ is spontaneous exothermic conversion. However, the rate‐limiting step in the total discharge process depends on largest increase of Gibbs free energy.^[^ ^7^, ^48^ ^]^ Notably, the formation of Li_2_S from Li_2_S_2_ needs the largest positive Gibbs free energy for both Ni‐HAB@CNT and CNT, which is 1.17 eV on CNT and 0.57 eV on Ni‐HAB@CNT. The lower Gibbs free energy on Ni‐HAB@CNT indicates that the S reduction is more thermodynamically favorable.

In addition, the activation energy barrier of Li_2_S decomposition was also examined to evaluate the initial charging process.^[^ ^49^ ^]^ Figure 6e and Figure s24 (Supporting Information) exhibit the initial state, final state, and transition state structure moles of Li_2_S decomposition on Ni‐HAB@CNT and CNT and corresponding energy change profiles. The calculated Li_2_S decomposition activation energy on the surface of the Ni‐HAB@CNT is just 1.05 eV, which is much lower than that of CNT (2.03 eV). As a result, Ni‐HAB@CNT can greatly reduce the decomposition energy barrier of Li_2_S, facilitating oxidation reaction kinetics, and thus speeding up the conversion from Li_2_S to LPSs in the following charge process.^[^ ^6^, ^28^ ^]^ Meanwhile, the Li_2_S oxidization was also examined by three‐electrode linear sweep voltammetry (LSV) technique (Figure S25, Supporting Information), in which AgCl/Ag, platinum sheet, and Li_2_S/methanol solution served as reference electrode, counter, and electrolyte, respectively. Figure 6f clearly reveals that the Ni‐HAB@CNT electrode delivers the lowest onset potential of −0.45 V and the largest current response compared with those of CNT (−0.41 V) and Ni‐HAB (−0.41 V), respectively, demonstrating the great kinetic enhancement. This conclusion can be further supported by the Tafel plots. As shown in Figure 6g, The minimal Tafel slope of 71 mV dec^−1^ can be obtained for Ni‐HAB@CNT electrode compared with that of CNT (163 mV dec^−1^) and Ni‐HAB electrodes (158 mV dec^−1^), respectively. These results are consistent with previous Li_2_S dissolution tests. All the aforementioned improvements in sulfur reaction kinetics can be attributed to the synergistic effect between Ni‐HAB and CNT. On the one hand, the highly metallic Ni‐HAB and conductive CNTs endow the catalytic interface with excellent electron transport properties. On the other hand, benefiting from the 2D structure of c‐MOF, the enriched unsaturated Ni atoms are fully exposed, leading to durable adsorption and catalytic activity.

**Figure 7:**
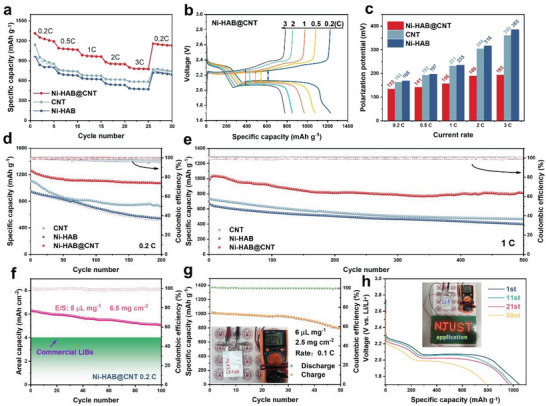
a) Rate performance of various modified separators at different specific currents; b) galvanostatic charge–discharge profiles of LSBs based on Ni‐HAB@CNT modified separators under different C‐rates; c) polarization potentials of various modified separators at different rates; d) cycling performance at 0.2 C; e) long‐term cycling stability at 1 C; f) cycle performance of Ni‐HAB@CNT separator with high sulfur loading at 0.2 C; g) electrochemical performances of the pouch cells with sulfur loading of 2.5 mg cm^−2^ and E/S ratio of 6 µL mg^−1^ at 0.1 C; the inset in (g) shows the digital photo of the fresh pouch cell and corresponding initial voltage; h) charge–discharge profiles of the pouch cell. Inset shows the digital photograph of the number of 67 LED lamps powered by Ni‐HAB@CNT pouch cell.

The electrochemical performance of various modified separators at different current density was tested in coin‐cell configuration. As shown in **Figure** **7**a, the cell with Ni‐HAB@CNT modified separator exhibits the best rate performance of 1310, 1089, 985, 881, and 799 mAh g^−1^ at a current density of 0.2, 0.5, 1, 2, and 3 C, respectively, significantly higher than those of CNT (945, 763, 682, 623, and 584 mAh g^−1^, respectively) and Ni‐HAB (953, 705, 627, 561, and 476 mAh g^−1^, respectively). When the current density is reduced to 0.2 C again, a reversible capacity of 1156 mAh g^−1^ can be obtained, demonstrating the high rate stability and fast reaction kinetics. The corresponding galvanostatic charge–discharge (GCD) profiles are given in Figure 7b and Figure S26a,b (Supporting Information). All the GCD curves exhibit two typical discharge plateaus and one charge plateau, which is consistent with the previous CV results and the multi‐step S reaction mechanism. In lithium–sulfur batteries, the discharge dip corresponds to the overpotential required for the nucleation and initial growth of Li_2_S.^[^ ^50^ ^]^ The overpotential magnitude is positively correlated with electrolyte viscosity, which is increased by the dissolution of polysulfides.^[^ ^51^ ^]^ As shown in Figure s26 c (Supporting Information), Ni‐HAB@CNT exhibits enhanced conversion of S_8_ to Li_2_S_4_ compared to Ni‐HAB and CNT (Lager Q1 value), resulting in a slight increase in the overpotential for Li_2_S nucleation. The exciting thing is that the polarization potential of the cell with Ni‐HAB@CNT modified separator is the smallest at all current densities (Figure 7c), along with the highest Q2/Q1 ratio (Figure S26d, Supporting Information). In particular, the polarization potential of the cell with Ni‐HAB@CNT is only 193 mV at 3 C, which is much lower than that of CNT (360 mV) and Ni‐HAB (385 mV). Meanwhile, the Ni‐HAB@CNT modified separator exhibited strong affinity to the electrolyte (Figure S27 and Videos S1–S3, Supporting Information), which facilitated faster lithium ion transport at the interface between the separator and electrodes, resulting in the lowest charge transfer impedance among all the cells with different separator (Figure S28, Supporting Information). Such remarkable rate performance and low polarization validate that the Ni‐HAB@CNT modified layer is effective in accelerating redox kinetics of LPSs and enhancing electrochemical cyclability.

As depicted in Figure 7d, the Li‐S cell with Ni‐HAB@CNT modified separator manifests superior cycling performance. After 200 cycles at 0.2 C, the reversible capacity of 1070 mAh g^−1^ with capacity retention of 85.2% can be maintained, and corresponding coulomb efficiency is close to 100%. The capacity decay rates and rate performance are superior to most other state‐of‐the‐art MOF‐based modified separators for LSBs (Table S2, Supporting Information). This can be ascribed to the smaller steric contact hindrance and increased delocalized electron density arising from the unique planar tetracoordinate structure in Ni‐HAB c‐MOFs, compared to other tetrahedral or octahedral coordination structures. In contrast, the cells with Ni‐HAB or CNT modified separators show a significant capacity fading. In addition, the long‐term cycle test of the cell with different separators was further measured at 1 C (Figure 7e). The cell with Ni‐HAB@CNT modified separator exhibits excellent cycling stability. A capacity of 804 mAh g^−1^ can be maintained with low capacity decay (per cycle) and nearly 100% coulombic efficiency even after 500 cycles. It should be noted that the capacity of Ni‐HAB@CNT batteries fluctuates between 795 and 810 mAh g^−1^ after 400 cycles, which can be attributed to variations in ambient temperature. In comparison, the cells with CNT and Ni‐HAB separators suffer from severe capacity decay with lower capacity retention of 463 and 399 mAh g^−1^ after 500 cycles, respectively.

In addition, postmortem analysis was performed by disassembling the cycled cells. Figure S29 (Supporting Information) displays the postmortem analysis of cycled Li metal anodes. Notably, the Li anode with Ni‐HAB/PP exhibits a rough and porous surface morphology (Figure S29a, Supporting Information). The highly aggregated Ni‐HAB particles fail to form a uniform coating on the separator surface, thus limiting its ability to suppress the diffusion of LPSs. The migration of LPSs results in the corrosion of the Li anode and detrimentally impacts its cycling performance, as demonstrated by the increased S signal observed in the EDS plot (Figure s29 d, Supporting Information). CNT facilitates a uniform coating layer on the separator surface, resulting in a smoother surface of the Li metal anode (Figure S29b, Supporting Information) and reduced S signal intensity (Figure S29e, Supporting Information), indicative of effective suppression of polysulfide migration. The cell containing Ni‐HAB@CNT/PP exhibits a smooth surface with minimal sulfur content, without severe pulverization and Li dendrite formation (Figure S29c,f, Supporting Information). Figure S30 (Supporting Information) shows the SEM images of cathode and separator cross‐sections of the cell with Ni‐HAB@CNT/PP. The cathode electrode was observed to maintain a porous structure without significant sulfur aggregation. Furthermore, Ni‐HAB@CNT modified layer exhibited a marginal increase in thickness while maintaining its overall structure, implying the stability of Ni‐HAB@CNT. These analyses further confirm the enhanced interaction between LPSs and catalysts achieved by Ni‐HAB@CNT, facilitating their conversion and effectively inhibiting LPSs migration. Furthermore, the impact of the modified separator layer on the lithium anode was assessed through the Li‐Li symmetrical cells. As shown in Figure S31 (Supporting Information), the commercial PP separator (without Ni‐HAB@CNT) demonstrates a large and fluctuating polarization overpotential exceeding 90.0 mV at a current density of 4 mA cm^−2^. In comparison, the cell employing Ni‐HAB@CNT/PP separator exhibits stable stripping/plating behavior and maintains a low overpotential of ≈40 mV. The results verify that the Ni‐HAB@CNT modified layer can effectively regulate the even distribution of lithium‐ion flux due to its uniform active sites and well‐tailored pore structure, resulting in a stable Li striping/plating process.

High sulfur area capacity and low electrolyte usage are important indicators to achieve the commercialization of LSBs.^[^ ^10^ ^]^ In general, under high sulfur loading conditions, the significant dissolution of polysulfides results in a sharp increase in electrolyte viscosity, leading to sluggish reaction kinetics.^[^ ^52^ ^]^ In this case, the rate‐determining step in lithium–sulfur batteries is the conversion of liquid‐phase Li_2_S_4_ to solid‐phase Li_2_S_2_/Li_2_S. Meanwhile, once the conductive substrate is covered by the early‐formed solid sulfides, it can self‐catalyze the reduction of Li_2_S_4_, thus enabling the continuous discharge reaction.^[^ ^53^ ^]^ Therefore, the key to achieving stable operation of the battery under high sulfur loading is to reduce the energy barrier for the formation of solid‐phase sulfides. Ni‐HAB@CNT can efficiently catalyze this step due to its excellent electrical conductivity and abundant Ni‐N active sites (Figures 5b and 6b–d). It should be noted that in high‐capacity sulfur cathode batteries, the thickness of the Ni‐HAB@CNT modification layer remained unchanged. As shown in Figure 7f, the cell with Ni‐HAB@CNT separator delivered discharge capacities of 6.29 mAh cm^−2^ at 0.2 C with a high sulfur loading (6.5 mg cm^−2^) and low E/S ratio (5 µL mg^−1^). As the cycling continued, the reversible capacities maintain 5.10 mAh cm^−2^ after 100 cycles, corresponding to a high capacity retention of about 81.1%, which is significantly superior to that of the commercial Li‐ion cell (≈4 mAh cm^−2^).

Besides the impressive coin‐cell performance, a single‐electrode pouch cell (4.3 × 5.6 cm) with Ni‐HAB@CNT modified separator shows a high initial potential and high initial capacity of 1015 mAh g^−1^ at 0.1 C with a sulfur loading of 2.5 mg cm^−2^ (Figure 7g). After 50 cycles, the cell can still deliver a quite stable and high reversible capacity of 791 mAh g^−1^ and maintain the typical discharge–charge plateaus and tolerable polarization (Figure 7h), indicative of a high sulfur utilization and a significant catalytic activity even with the low electrolyte usage (E/S = 6 µL mg^−1^). Accordingly, based on the high total sulfur loading, the assembled Li‐S pouch battery can keep the number of 67 led lamps lit for over 24 h (inset of Figure 7h and Figure S32, Supporting Information).

Overall, the Ni‐HAB@CNT modified separator endows the Li‐S cell with excellent electrochemical performance, which can be ascribed to the significant synergistic effects between Ni‐HAB and CNT. First, there is a superior electrical conductivity induced by the delocalization of electrons in the π–d conjugated Ni‐HAB c‐MOF and CNT, improving the sulfur utilization. Second, the Ni‐HAB c‐MOFs feature well‐tailored sub‐nanometer pore sizes, facilitating selective permeation of lithium ions and effectively mitigating the diffusion of LPSs. Finally, the abundant Ni‐N_4_ centers with low steric contact hindrance can serve as efficient active sites to catalyze S redox reactions. Therefore, it is expected to definitively promote the practical process of LSBs.

## 3 Conclusion

In summary, we have rationally designed a unique CNT‐supported 2D Ni‐HAB c‐MOF as PP separator wrapper layer by using the smallest π‐conjugated ligand HAB through a one‐step in situ growth strategy. The highly ordered engineered subnanometer pores (≈8 Å), exceptional conductivity derived from abundant delocalized electrons, and low steric contact resistance resulting from planar tetracoordination make Ni‐HAB@CNT as an ideal LPS barrier for achieving high‐sulfur‐loading and pouch LSBs. Besides, as revealed by the theoretical calculations and in situ characterizations, the high‐density Ni‐N coordination centers enhanced sulfur affinity and thus facilitated LPSs redox kinetics. As a result, the assembled LSBs with Ni‐HAB@CNT modified separator delivered impressive cycle performance (a low capacity‐fade rate of 0.041% cycle^−1^ after 500 cycles; a high sulfur loading of 6.5 mg cm^−2^ corresponding to the area specific capacity of 6.29 mAh cm^−2^). This work demonstrates the 2D Ni‐HAB c‐MOF can effectively suppress shuttle effects and accelerate the conversion of S related species.

## Conflict of Interest

The authors declare no conflict of interest.

## Supporting information

Supporting Information

Supplemental Video 1

Supplemental Video 2

Supplemental Video 3

## Data Availability

The data that support the findings of this study are available in the supplementary material of this article.
