# Supplementary material for: High Crystallinity 2D π–d Conjugated Conductive Metal–Organic Framework for Boosting Polysulfide Conversion in Lithium–Sulfur Batteries
Source: Adv Sci (Weinh). 2023 Jul 28;10(27):2302518. doi: 10.1002/advs.202302518 (PMC10520645; doi:10.1002/advs.202302518)
Supplement: Supplementary file 1 — Supporting Information [file ADVS-10-2302518-s001.pdf]

ADVANCED SCIENCE
Open Access

Supporting Information

for *Adv. Sci.*, DOI 10.1002/advs.202302518

High Crystallinity 2D π–d Conjugated Conductive Metal–Organic Framework for Boosting Polysulfide Conversion in Lithium–Sulfur Batteries

Tong Guo, Yichen Ding, Chang Xu, Wuxin Bai, Shencheng Pan, Mingliang Liu, Min Bi, Jingwen Sun, Xiaoping Ouyang, Xin Wang, Yongsheng Fu and Junwu Zhu**

Supporting Information

High Crystallinity Two-Dimensional π-d Conjugated Conductive Metal-Organic Framework for Boosting Polysulfide Conversion in Lithium-Sulfur Batteries

Tong Guo, Yichen Ding, Chang Xu, Wuxin Bai, Shencheng Pan, Mingliang Liu, Min Bi, Jingwen Sun, Xiaoping Ouyang, Xin Wang, Yongsheng Fu, Junwu Zhu**

EXPERIMENTAL SECTION

Preparation of Ni-HAB@CNT Composites.

The carbon nanotubes (CNTs, Jiangsu Xianfeng Nanomaterials Technology Co., Ltd.) were treated in a mixture of concentrated H_2SO_4/HNO_3 (3:1) in an oil bath at 100 °C for 4 h before being used. To synthesize the Ni-HAB@CNT, CNTs (50 mg) were dispersed in deoxygenated 1-Methyl-2-pyrrolidinone (NMP, 25 mL) by ultra-sonication for 1 h in a three-necked round bottom flask. $Ni(NO_3)_2{\cdot}6H_2O$ (218 mg, 0.75 mmol) was dissolved in deoxygenated NMP (15 mL) and then added to the above CNTs solution under vigorous stirring for 0.5 h at room temperature. The above-mixed solution was named solution A. Protonated hexaaminobenzene (HAB·3HCl, 139 mg, 0.5 mmol) was dissolved into deoxygenated NMP (15 mL) to form solution B. Next, solutions B and $NH_3{\cdot}H_2O$ (75 μL) were added to solution A drop by drop. The mixture was heated to 140 °C for 2 h. After being cooled to room temperature, the final product was gathered by centrifugation, washed with ethanol and water at least three times, and freeze-dried for 24 h.

Synthesis of modified separators and sulfur cathodes

The Ni-HAB@CNT modified separator was prepared by the same filtration method. Briefly, Ni-HAB@CNT was dispersed by sonication in ethanol to get a homogeneous solution, and the solution was then vacuum filtered on a commercial separator (Celgard 2400) to obtain the modified separators. The mass loading was controlled at around 0.15 mg cm^{-2}. As shown in Table S3, the modification layer prepared in this study exhibited the lowest thickness and mass density, which can effectively reduce the adverse effects of the modified layer on energy density. For comparison, Ni-HAB and CNT modified separators were also prepared respectively by the same process. Eventually, all the coated separators were cut into discs with a diameter of 19 mm. The carbon nanotube/sulfur (CNT/S) composites were prepared by a melt diffusion method. Typically, CNT and sublimed sulfur powder were well ground in a weight ratio of 1:4 and heated at 155 °C for 10 h.

Material characterization

The morphologies of the materials were characterized by field-emission scanning electron microscopy (FESEM, JEOL JEM7800F Prime) equipped with energy-dispersive X-ray spectroscopy (EDS), transmission electron microscope (TEM; JEOL, JEM-2100, operated at 200 kV), and high-resolution TEM (HRTEM) (FEI Talos F200X G2) equipped with EDS (FEI SuperX G2). The crystal structure of materials was characterized by X-ray powder diffractometer (XRD, Miniflex-600W, Rigaku Corporation, Tokyo, Japan) with Cu K αradiation (λ = 1.54 Å). X-ray photoelectron spectra (XPS) were obtained on the Thermo Fisher Nexsa XPS system. Raman spectra were obtained with a Renishaw Raman Microprobe. As shown in Figure S33, thermogravimetric analysis (TGA, TA Instruments TGA-Q50) was employed to record the ratio of sulfur in CNT/S (80 wt %). It is

noteworthy that the distribution form of sulfur changes after the thermal fusion composite with CNT. The sulfur undergoes from the original bulk shape to a spatial distribution around the CNT, which reduces sulfur aggregation, leading to a decrease in its thermal stability but an increase in its electrochemical activity, compared to the original bulk sulfur. The electrical conductivity of Ni-HAB was measured by a four-point probe method at room temperature (~ 298 K). The Ni-HAB pellet (150 ~ 170 μm) was made by cold isostatic pressing at 80 MPa for 15 min using commercial pressing equipment (SHIMADZU).

Electrochemical performance measurements

To fabricate the sulfur cathode, the as-synthesized CNT/S composites were mixed with super P and PVDF powder in a weight of 8: 1: 1 in NMP and stirred to form uniform slurry which was then cast onto an Al foil current collector and dried at 60 °C overnight. The dried cathodes were cut into disks with a diameter of 12 mm and the sulfur mass loadings were about 1.5 ~ 2 mg cm^{-2}. A lithium metal disk (diameter = 15.6 mm, thickness = 450 μm) was used as the anode. The electrolyte was 1.0 M LiTFSI in DOL and DME (1: 1 by volume) with 2 wt% $LiNO_3$ additive. It should be noted that the main function of the $LiNO_3$ additive is to build a solid electrolyte interface (SEI) on the surface of the lithium metal anode, which helps to improve the coulombic efficiency of the battery (Figure S33 a-c). Furthermore, the contribution of $LiNO_3$ to the battery's discharge capacity is negligible in the voltage range of 1.7-2.8V (Figure S33 b-d). The CR2032 coin cells were assembled in an argon-filled glove box (< 1 ppm of O_2 and H_2O) and the ratios of electrolyte to S were controlled to be 15 μL mg^{-1}. Galvanostatic charge/discharge measurements were performed on a LAND CT2001A testing system

with a voltage window of 1.7−2.8 V under various C-rates (1 C = 1672 mAh g^{-1}, based S). The autolab PGSTAT302N (Metrohm, Switzerland) electrochemical workstation was used to collect the cyclic voltammetry (CV) and electrochemical impedance spectroscopy (EIS) spectra.

Li || Li symmetric cells assembly

All Li–Li symmetric cells were assembled in an Argon-filled glove box (< 1 ppm of O_2 and H_2O). The electrolyte was kept in line with those of conventional Li-S batteries, ensuring a controlled total volume of 30 μL. The commercial PP (Celgard 2500) and Ni-HAB@CNT/PP were used as separators, respectively.

Preparation of Li_2S_6 solution and adsorption tests

To prepare Li_2S_6 solutions (5 mM), sulfur and Li_2S were dissolved in DME/DOL (v: v = 1:1) solution at a molar ratio of 5:1. The solution was continuously stirred at 60 °C for 12 hours in a glove box. To evaluate the absorption ability of polysulfides, equal amounts of CNT, Ni-HAB, or Ni-HAB@CNT were added to 3 mL of the Li_2S_6 solution prepared as described above. The samples were then soaked in the solution for 6 h, after which ex-situ UV-visible absorption spectra were measured. All of the above operations were conducted in an argon-filled glove box.

Shuttle current measurements

For the shuttle current measurement, cells with a sulfur loading of ~2 mg cm^{-2} were assembled without $LiNO_3$ additive. Typically, the cells were charged and discharged 2 cycles at the current density of 0.2

C before galvanostatically charged to 2.8 V. Then the cells were discharged to 2.38 V and switched to the potentiostatic mode during which the current reached a steady-state value. This steady-state current was recorded as the shuttle current.[1]

Li_2S nucleation measurements

For the study of liquid-solid conversion kinetics, the Ni-HAB@CNT materials (or Ni-HAB, CNT) were dissolved in isopropanol and then dropped onto round carbon paper disks (12 mm), the total mass loading was controlled around 1 mg. 25 μL Li_2S_8 catholyte (0.2 M Li_2S_8, 1 M LiTFSI in tetreglyme) and 20 μL electrolytes without Li_2S_8 were added to the cathode and anode sites, respectively. The cells were first discharged to 2.06 V under a constant current of 0.112 mA and then kept potentiostatically at 2.05V until the current dropped below 10^{-5} A. The nucleation capacity of Li_2S can be calculated by the integral area of the plotted curve through Faraday's Law.[2]

Li_2S dissolution measurements

To evaluate the dissolution behavior of Li_2S, fresh coin cells were assembled and subjected to galvanostatic discharge at 0.10 mA until reaching 1.7 V. Subsequently, the cells underwent an additional galvanostatic discharge cycles at 0.01 mA to reach 1.80 V for full transformation of sulfur species into solid Li_2S. Then the cells were then potentiostatically charged at 2.35 V to facilitate the dissolution of Li_2S into soluble polysulfides until the charge current dropped below 0.01 mA.

Symmetric cell assembly and kinetic evaluation of polysulfide conversion

The symmetric cell used Ni-HAB@CNT (or Ni-HAB, CNT) as identical counter and working electrodes with a mass loading of 1 mg. 40 μL Li_2S_6 solution (0.2 M Li_2S_6 and 1 M LiTFSI in DOL/DME, v/v = 1/1) was used as the electrolyte. The CV measurements of the symmetric cells were performed with a voltage window between -1 to 1 V.

In-situ Raman and XRD spectroscopy measurements

The in-situ Raman and XRD devices were purchased from Beijing Scistar Technology Co., Ltd. A quartz window was used for laser passage for in-situ Raman. A hole (2 mm) was punched in the Li-metal anode to allow the laser to focus directly on the separator. The cells were run at a current density of 0.5 C. Raman signals were obtained on Renishaw Microprobe by a 532 nm laser. It should be noted that, in order to minimize the interference of the electrolyte with the optical path, the battery testing was conducted under lean electrolyte conditions (8 μL mg^{-1}). As for the in-situ XRD spectrum test, a beryllium window was used for X-ray penetration; Ultrathin Al foil (~6 μm) was used as current collector. The sulfur loading of each cell was about 3 mg to get better signal. The cells were run at a current density of 0.1 C for in-situ XRD. Continuous scanning during cell running with each scan measured at 15-40° in 0.02° incremental steps.

Lithium ion conductivity measurement

The lithium ion conductivity (σ) of the coin cell was calculated from the impedance data, using the formula:

$$\sigma = \frac{d}{R \cdot A} \qquad (1)$$

where d and A are the thickness and face area of the sample, respectively, and R is derived from the low intersection of the high frequency semi-circle on a complex impedance plane with the Z axis.

Based on the Arrhenius relationship between ionic conductivity and temperature:

$$\text{In}\ \sigma = \text{In}\ \sigma_0 - \frac{E_a}{RT} \qquad (2)$$

where R, T, $\sigma_{0,}$ and Ea are the gas constant (8.314 J K^{-1} mol^{-1}), the absolute temperature and the frequency factor (independent of T), and the activation energy of ion conduction, respectively. The apparent activation energy (Ea) of ionic conductivity was calculated according to the following formula:

$$E_a = -b \times R \qquad (3)$$

where b is the slope of the straight line obtained from the plot of lnσ against 1000/T.

Computational details

All calculations were performed using density functional theory (DFT) method implemented in Vienna ab initio simulation package (VASP).[3] Generalized gradient approximation (GGA) with the Perdew-Burke-Ernzerhof (PBE) functional is employed to treat the exchange-correlation energy.[4-5] The interaction between core and valence electrons was described by the projected augmented wave (PAW) potential. A plane-wave cutoff energy of 500 eV is adopted for the standard norm-conserving pseudopotentials. The Methfessel–Paxton smearing[6] with a smearing energy width of 0.10 eV and Γ-centered Monkhorst-Pack k-point meshes[7] for Brillion zone integration. Custom k-point grids of 2 × 2 × 1 were used for all surface structures. The electronic energy was considered self-consistent when the energy change was smaller than 10^{-5} eV. A geometry optimization was considered convergent when

the force change was smaller than 0.02 eV/Å. The climbing-image nudged elastic band (CI-NEB) method implemented in VASP was performed to investigate the transition state searches.[8] Charge transfer between the Ni-HAB@CNT (CNT) and the Li_2S_6 was determined by performing Bader analysis.[9]

In this work, The adsorption energy (E_{ads}) can be obtained by the equation (n = 1, 2, 4, 6, 8):

$$E_{ads} = E_{sub + Li_2S_n} - (E_{sub} + E_{Li_2S_n}) \quad (4)$$

where $E_{sub + Li_2S_n}$, E_{sub}, and $E_{Li_2S_n}$ are the energy of polysulfides-substrate, substrate, and polysulfide.

In lithium-sulfur batteries (LSBs), the overall sulfur redox reaction (SRR) of an S_8 molecule is a 16-electron process in the discharge reactions, which is accompanied by the formation of eight Li_2S molecules:[10]

$$S_8 + 16\ Li^+ + 16\ e^- \rightarrow 8\ Li_2S$$

The production process of a Li_2S molecule involves the following steps.[11-12]

$$*S_8 + 2\ Li^+ + 2\ e^- \rightarrow *Li_2S_8$$

$$*Li_2S_8 \rightarrow *Li_2S_6 + 1/4\ S_8$$

$$*Li_2S_6 \rightarrow *Li_2S_4 + 1/4\ S_8$$

$$*Li_2S_4 \rightarrow *Li_2S_2 + 1/4\ S_8$$

$$*Li_2S_2 \rightarrow *Li_2S + 1/8\ S_8$$

Where * represents an active site on the catalytic surface.

The free energy is calculated as follows: G = E + ZPE – TS, where G, E, ZPE, and TS are the free energy, total energy from DFT calculations, zero-point energy, and entropic contributions, respectively.

Note: band structure was calculated with HSE06 functional accuracy.

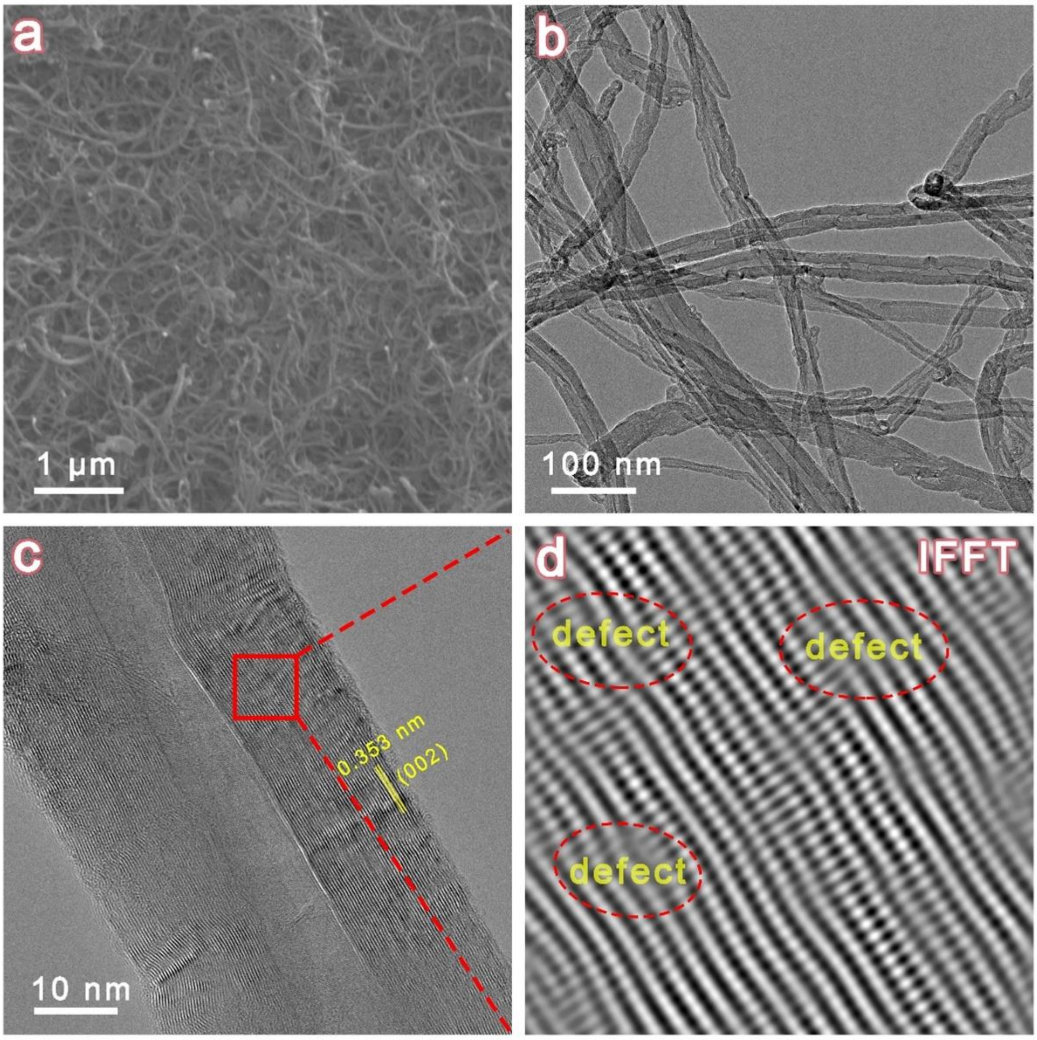

Figure S1. (a) FE-SEM, (b) TEM, and (c) HRTEM images of acid-treated CNTs; (d) IFFT spectrum were obtained from the red squared area in the HRTEM micrograph.

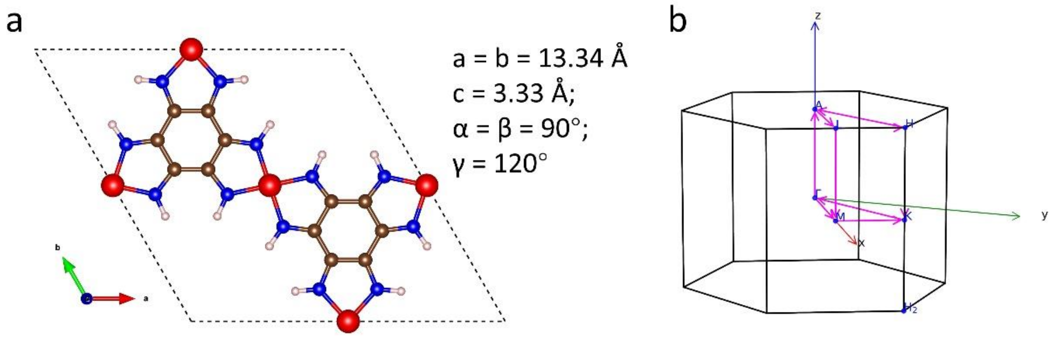

Figure S2. (a) The primitive cell geometric structure and parameters of optimized Ni-HAB bulk; (b) First Brillouin zone of Ni-HAB primitive cell. Path: Γ → M → K → Γ → A → L → H → A|L → M|H→K.

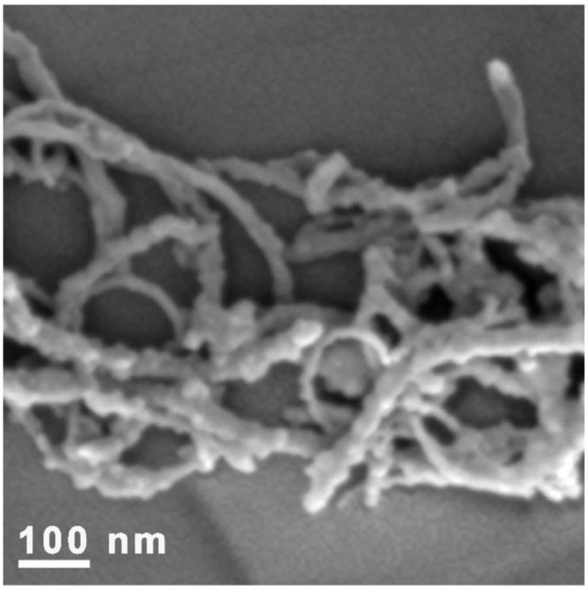

Figure S3. SEM image of Ni-HAB@CNT.

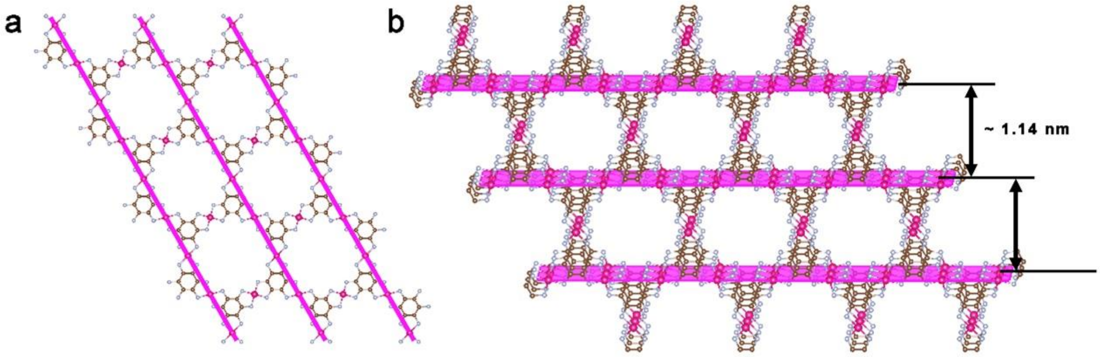

Figure S4. (a) top and (b) side views of {100} crystallographic plane of Ni-HAB based on DFT calculation results.

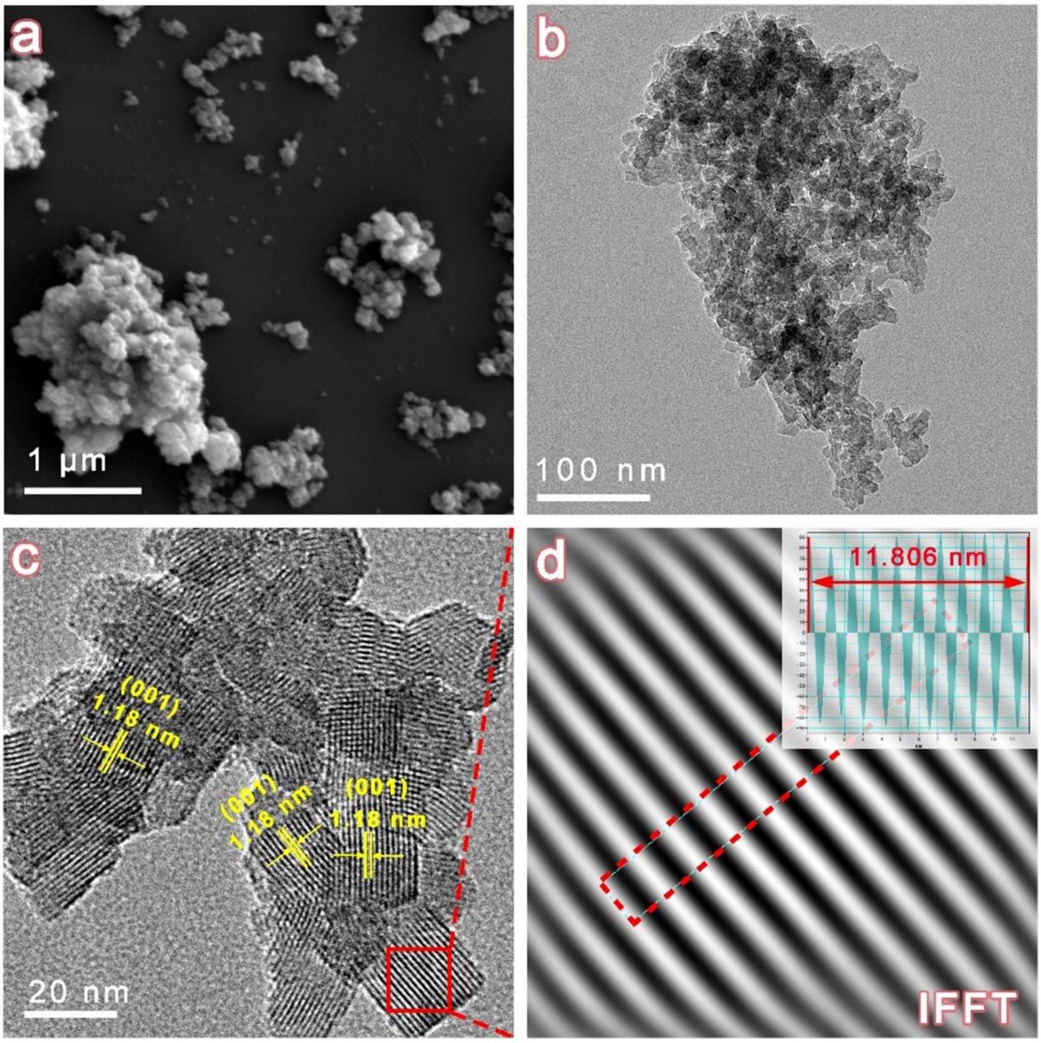

Figure S5. (a) FE-SEM, (b) TEM, and (c) HRTEM images of Ni-HAB nanoparticles; (d) IFFT spectrum were obtained from the red squared area in the HRTEM micrograph.

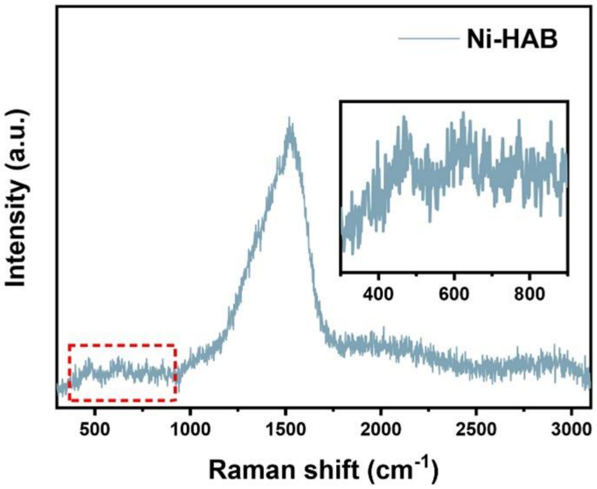

Figure S6. Raman spectrum of Ni-HAB.

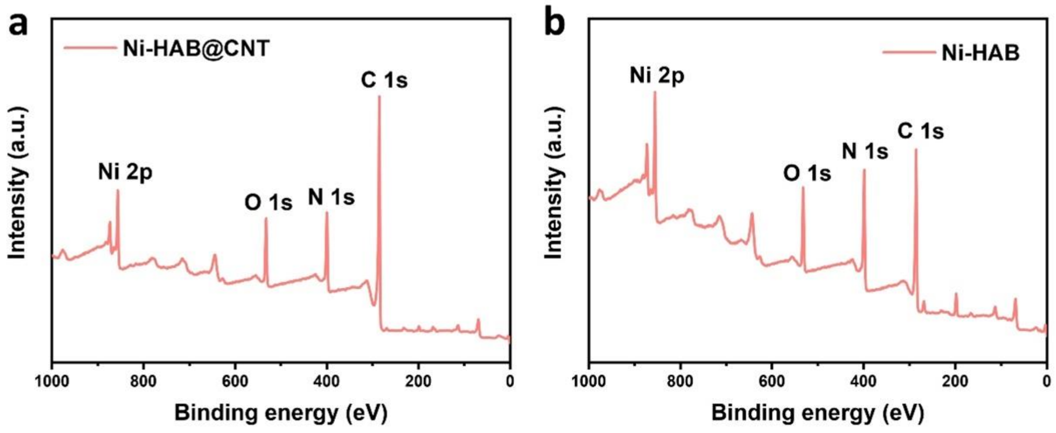

Figure S7. XPS survey spectrum of (a) Ni-HAB@CNT and (b) Ni-HAB.

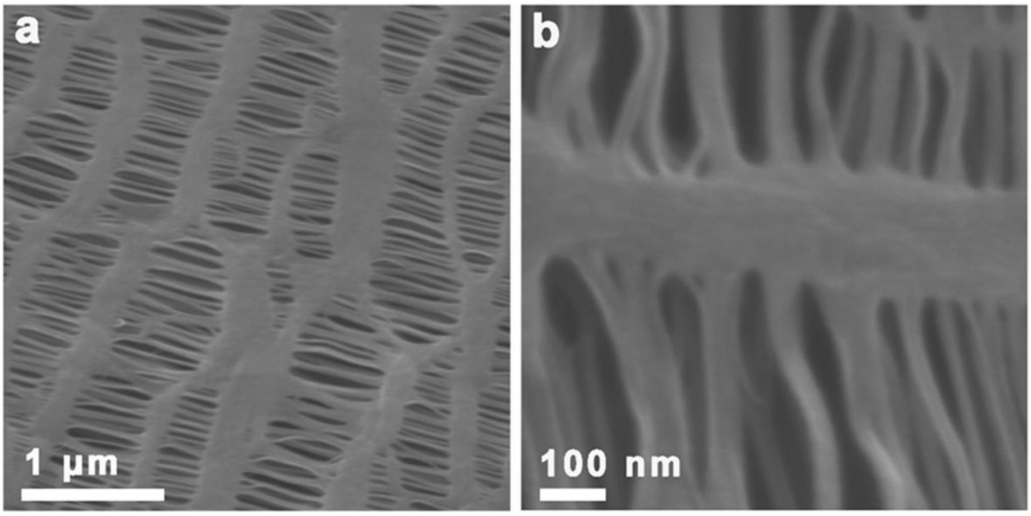

Figure S8. (a,b) SEM images of the PP separator surface at different magnifications.

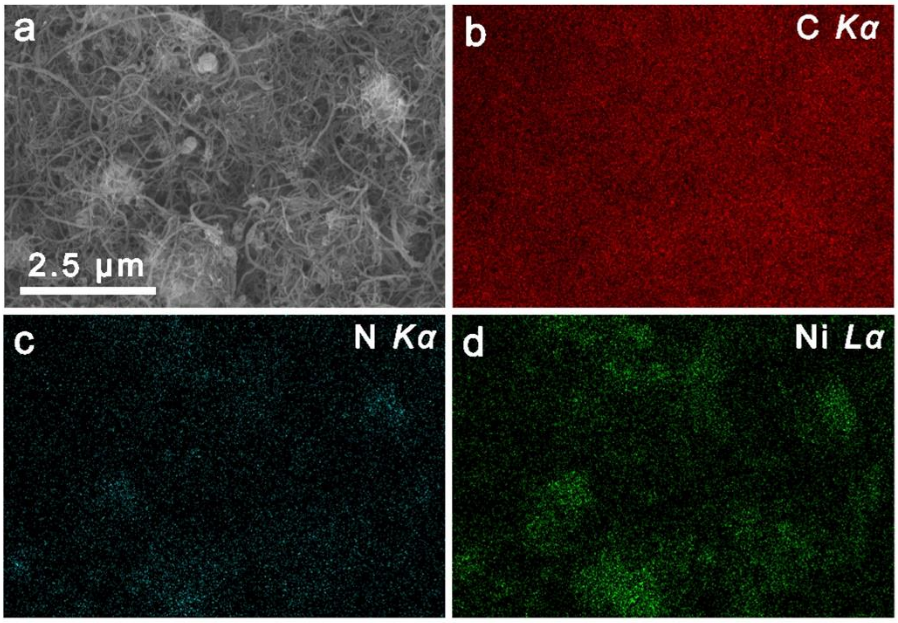

Figure S9. (a) Top-view SEM image and (b-d) corresponding element mapping of the Ni-HAB@CNT modified PP.

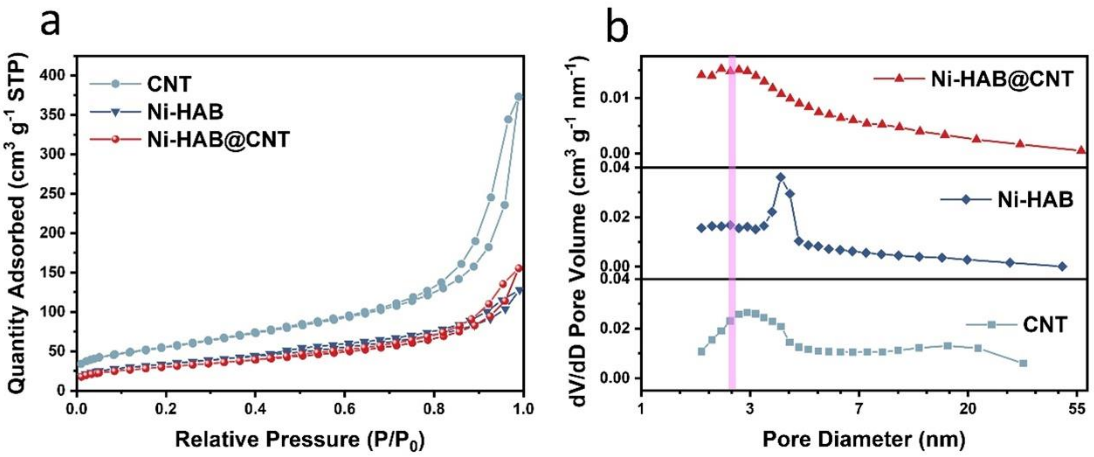

Figure S10. (a) N_2 adsorption-desorption isotherms at 77 K and (b) corresponding BJH pore-size distribution plots of CNT, Ni-HAB, and Ni-HAB@CNT.

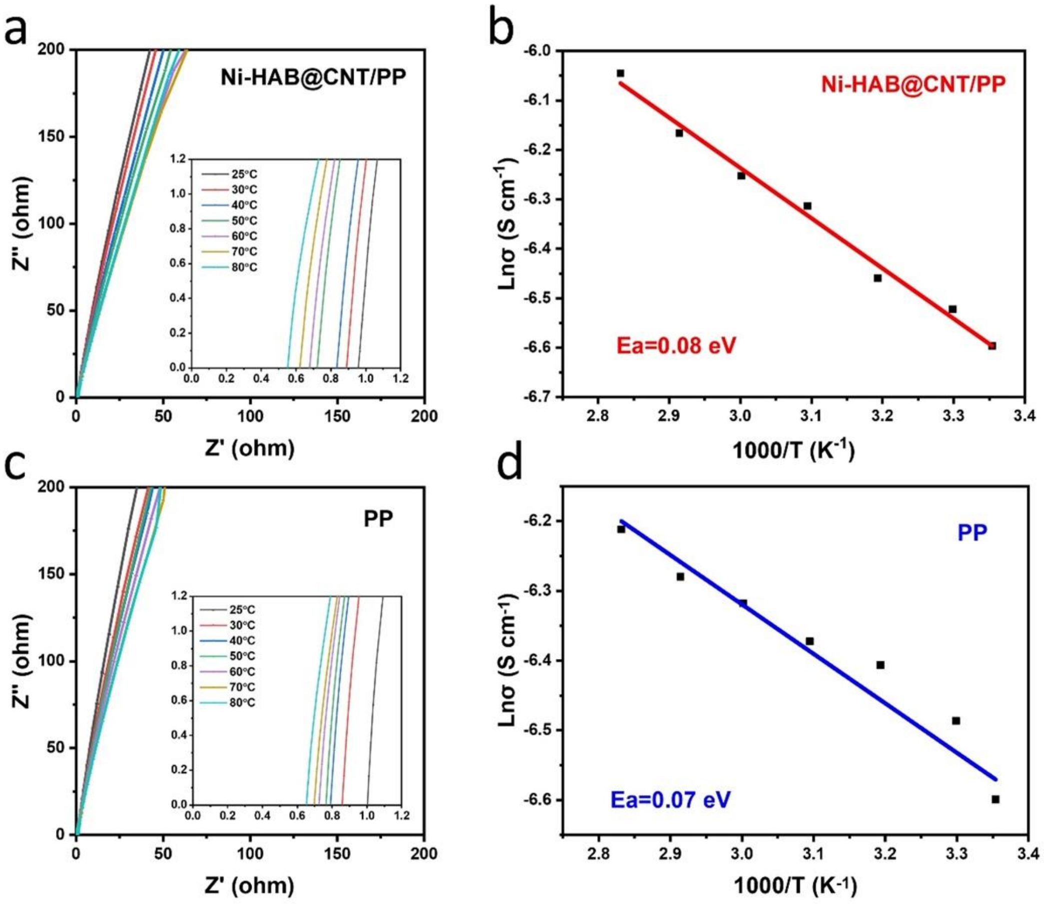

Figure S11. (a) Electrical impedance spectroscopy and (b) activation energy (E_a) of Ni-HAB@CNT/PP; (c) Electrical impedance spectroscopy and (d) activation energy (E_a) of PP.

The Li^+ conductivities of the Ni-HAB@CNT/PP and PP separators at 25 °C are 1.362×10^{-3} and 1.365×10^{-3} S cm^{-1}, respectively. These results indicate that the Ni-HAB@CNT barrier layer has no negative impact on the transport of Li^+.

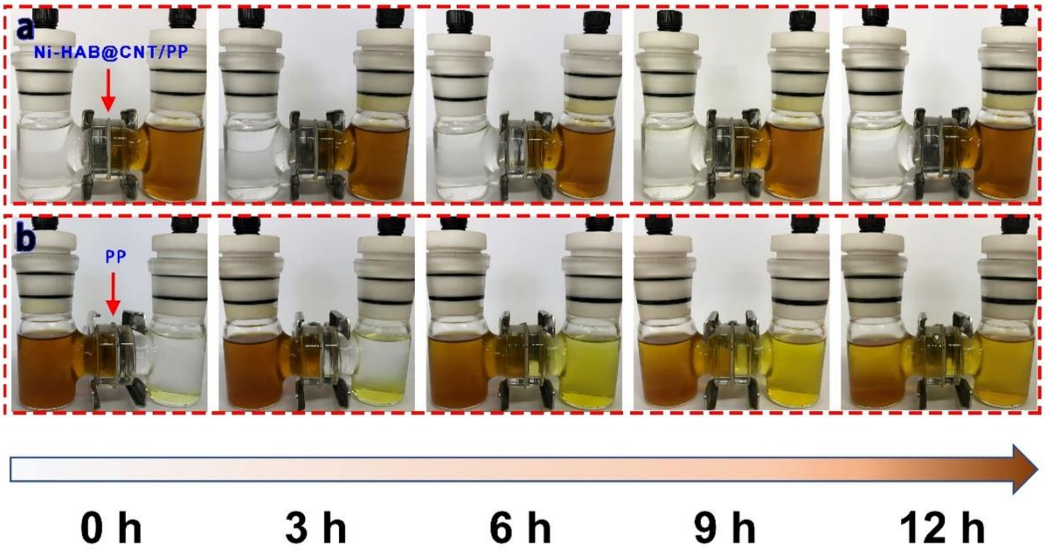

Figure S12. Permeation experiments with an H type of permeation for Ni-HAB@CNT modified PP (a) and PP (b).

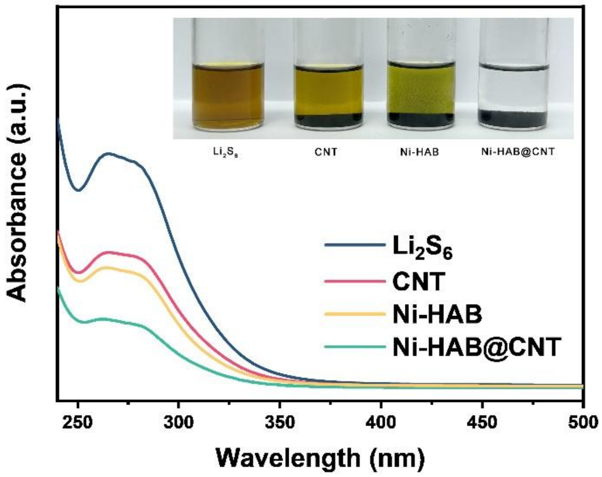

Figure S13. UV-vis absorption spectra of different samples after immersion in Li_2S_6 solution for 6 h. Inset: optical photograph of Li_2S_6 adsorption experiment.

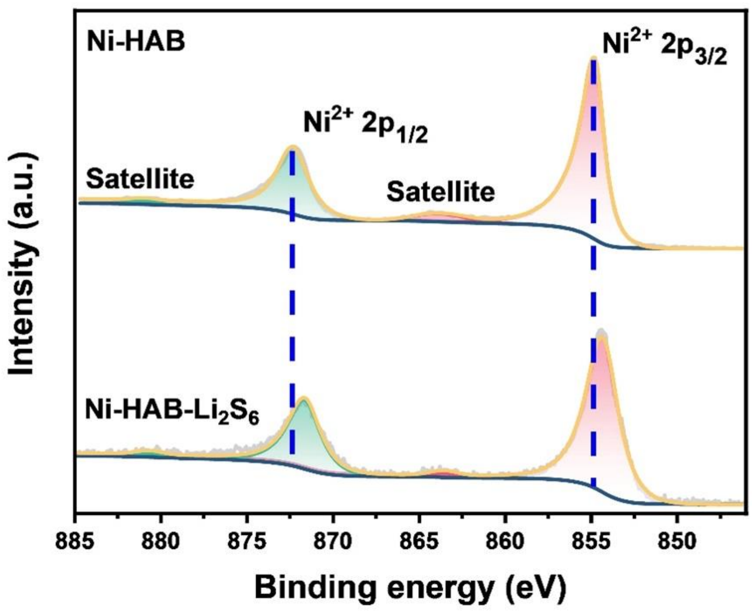

Figure S14. Ni 2p XPS spectrum of Ni-HAB before and after adsorption of Li_2S_6.

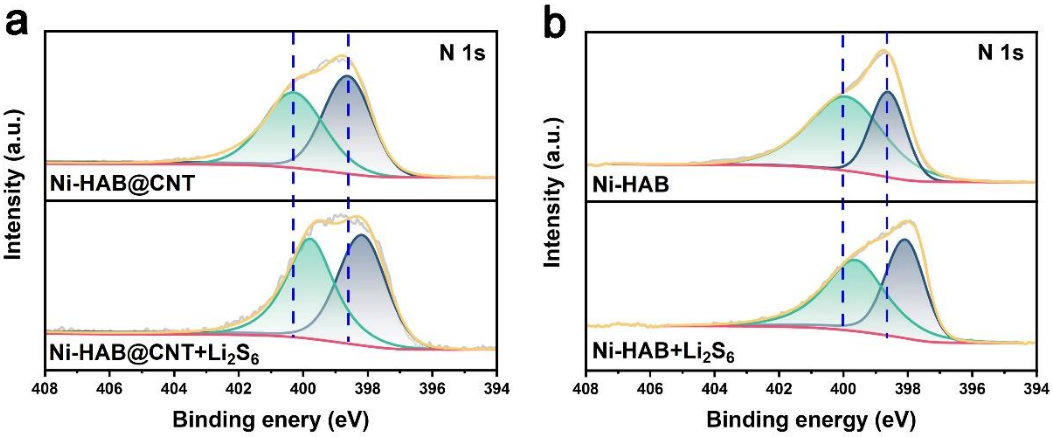

Figure S15. N 1s XPS spectra of Ni-HAB@CNT (a) and Ni-HAB (b) before and after adsorption of Li_2S_6.

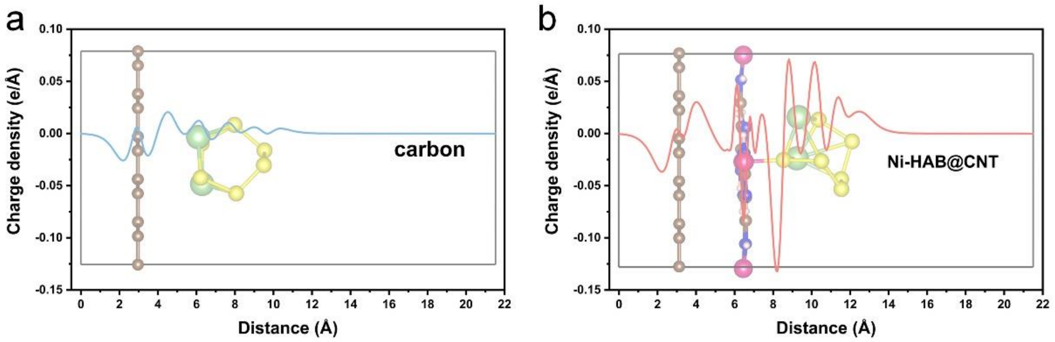

Figure S16. Planar average charge density difference along the z-direction (a) between Li_2S_6 molecule and carbon, (b) between Li_2S_6 and Ni-HAB@CNT.

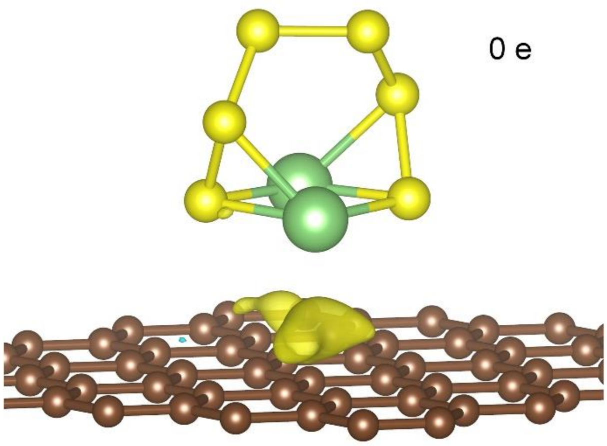

Figure S17. Charge density difference of Li_2S_6 adsorbed on CNT.

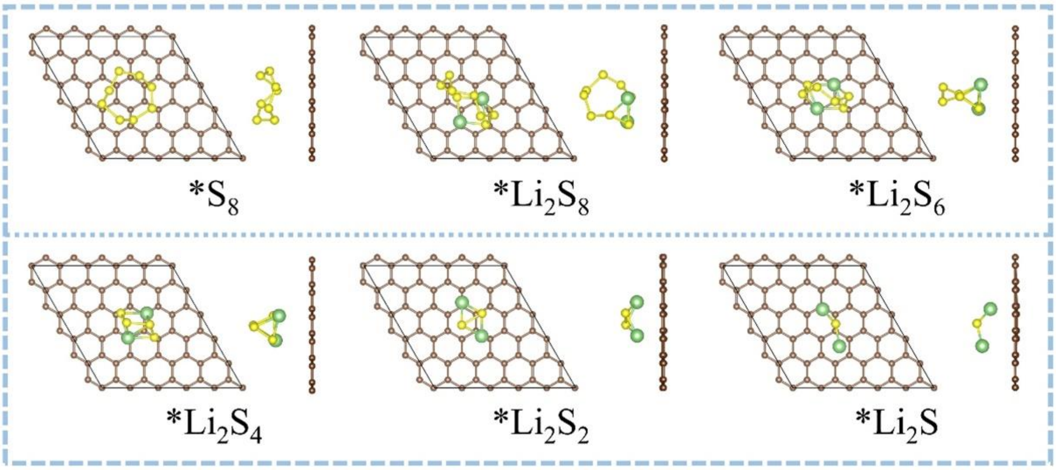

Figure S18. The optimized adsorption configurations of S_8 and LPSs species on the CNT surface. The atoms with different colors correspond to C (brown), S (yellow), and Li (green), respectively.

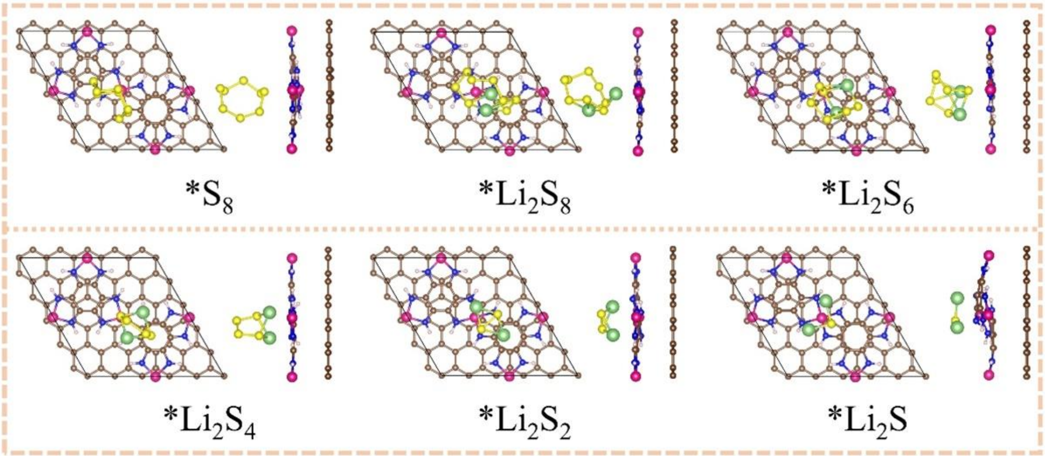

Figure S19. The optimized adsorption configurations of S8 and LPSs species on the Ni-HAB@CNT surface. The atoms with different colors correspond to C (brown), Co (pink), N (blue), S (yellow), and Li (green), respectively.

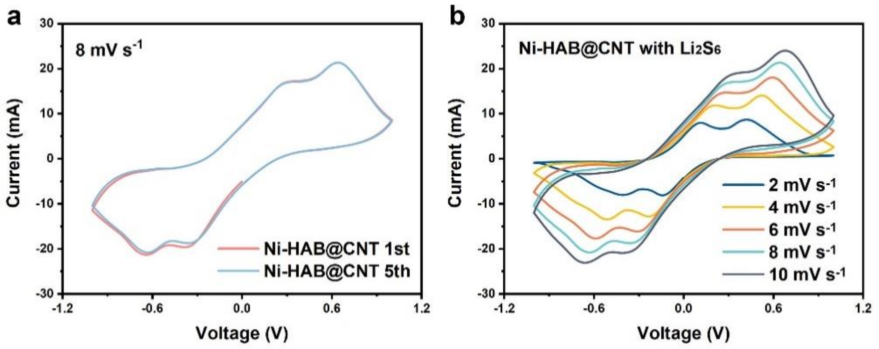

Figure S20. (a) CV curves of the symmetric cells with identical electrodes of Ni-HAB@CNT in an electrolyte with 0.2 M Li_2S_6 at 8 mV s^{-1}; (b) CV curves of the symmetric cell with identical electrodes of Ni-HAB@CNT at different scan rates.

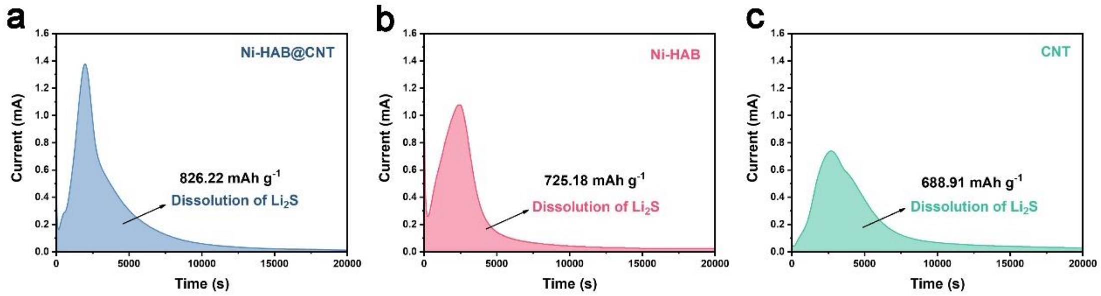

Figure S21. (a) Dissolution profiles of Li_2S with (a) Ni-HAB@CNT, (b) Ni-HAB, and (c) CNT to evaluate dissolution behaviors of Li_2S.

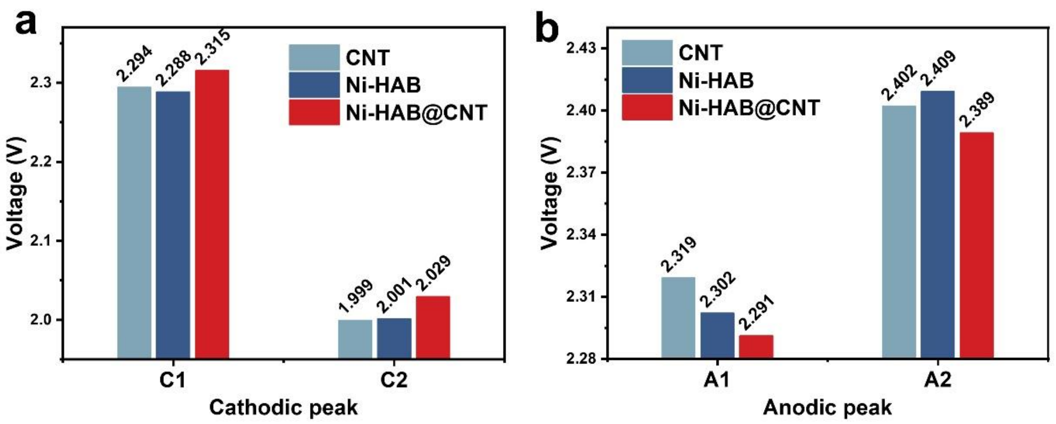

Figure S22. (a) Cathodic peak and (b) Anodic peak potentials of the Li-S coin cell with CNT, Ni-HAB, and Ni-HAB@CNT modified separator.

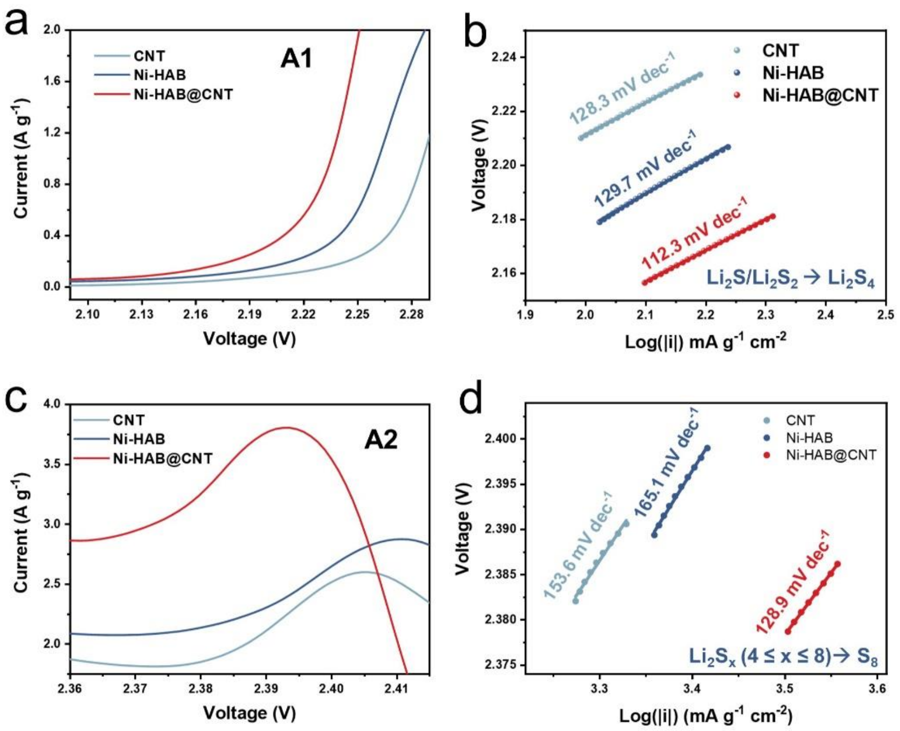

Figure S23. (a) the enlarged anodic peak in the range of 2.09 -2.29 V and (b) corresponding Tafel profiles; (c) the enlarged anodic peak in the range of 2.36 -2.41 V and (d) corresponding tafel profiles.

For the peak A1 ($Li_2S/Li_2S_2 \rightarrow Li_2S_4$), the Tafel slopes for Ni-HAB@CNT, Ni-HAB, and CNT are 112.3, 129.7, and 128.3 mV dec^{-1}, respectively (Figure S21a,b). For the peak A2 (Li_2S_x ($4 \leq x \leq 8$) $\rightarrow$ Li_2S_2/Li_2S), they are 128.9, 165.1, and 153.6 mV dec^{-1}, respectively (Figure S21c,d). These results demonstrate that Ni-HAB@CNT can accelerate the conversion rate of Li_2S to S_8 during the charging process, which is consistent with the dissolution of Li_2S.

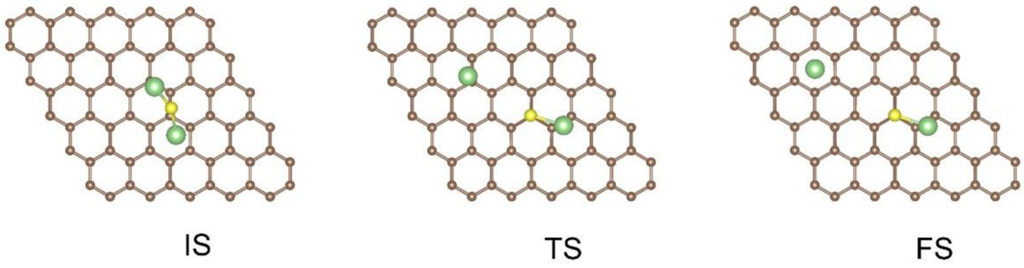

Figure S24. The optimized configurations of Li_2S decomposition on the CNT surface. The atoms with different colors correspond to C (brown), S (yellow), and Li (green), respectively.

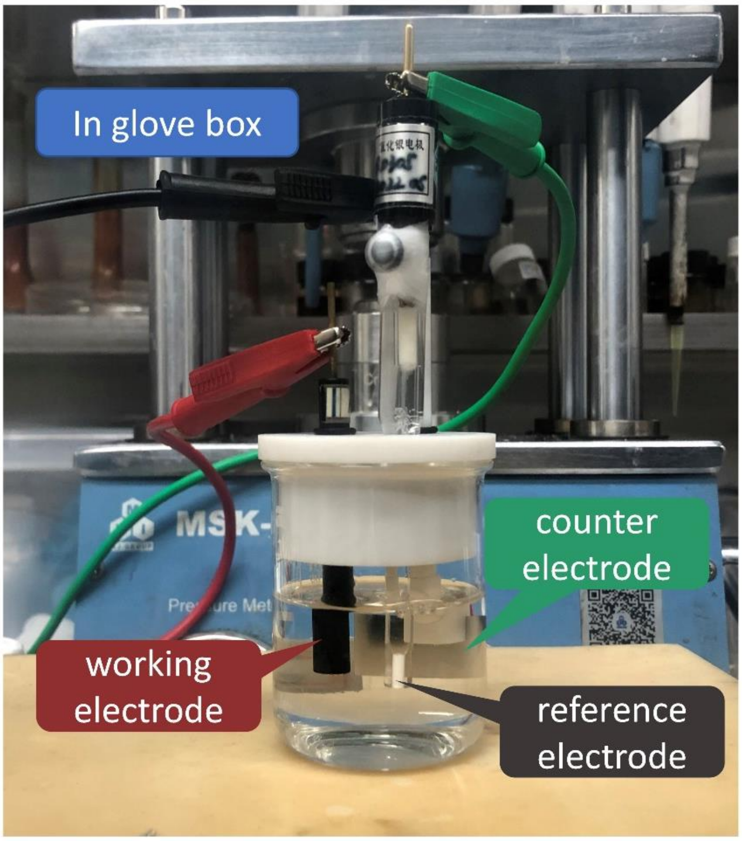

Figure S25. Digital photograph of the three-electrode LSV device for Li_2S decomposition in an Ar-filled glove box.

Note: As we know, Li_2S is not soluble in DOL/DME mixed solvent, so it is impossible to use DOL/DME mixed solvent to construct the contact interface between the catalyst and Li_2S. Methanol, which has a higher dielectric constant, can dissolve Li_2S without reacting with it. Therefore, using methanol as a solvent can establish a stable interface between the catalyst and Li_2S. Although this testing condition differs significantly from the actual operating conditions of the battery, it is still possible to qualitatively analyze the catalytic ability of different materials on the Li_2S oxidation process.

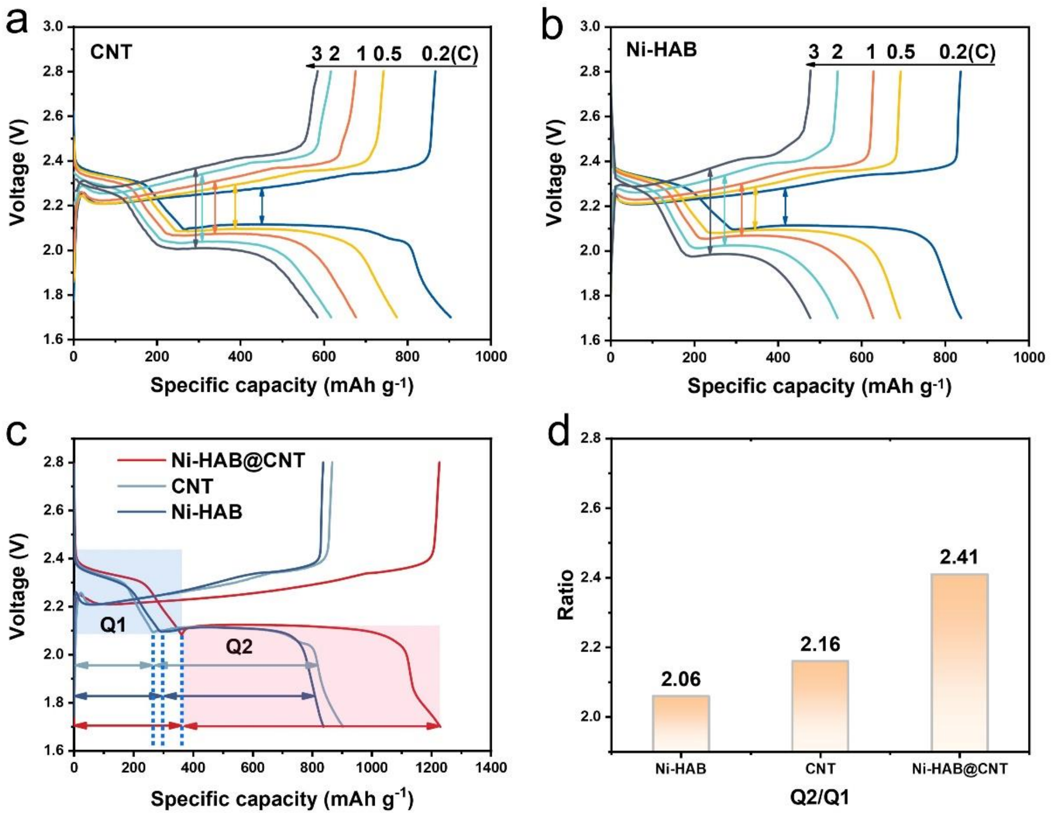

Figure S26. Galvanostatic charge-discharge profiles of LSBs based on (a) CNT and (b) Ni-HAB modified separator under different C-rates; (c) Galvanostatic charge-discharge profiles of Li-S batteries based on various materials at a current rate of 0.2 C; (d) The value Q2/Q1 obtained from charge-discharge curves of various materials.

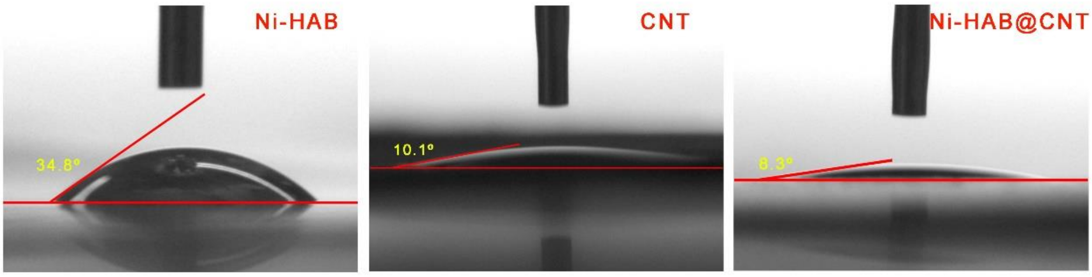

Figure S27. Contact angle measurement of Li-S electrolyte on the surface of the Ni-HAB, CNT, and Ni-HAB@CNT modified separators.

The electrolyte's wetting behavior on the separator is important in determining the electrochemical performance of a battery. Figure S26 shows photographs obtained by dropping the same amount of electrolyte onto the Ni-HAB modified separator, CNT modified separator, and Ni-HAB@CNT modified separator. At the interface between the electrolyte and the Ni-HAB@CNT modified separator, the contact angle between the electrolyte and the Ni-HAB@CNT modified separator interface is measured to be 8.3°, which is lower than that of the other interfaces. This indicates that the surface tension at the electrolyte/modified separator interface is lower, facilitating the wetting of the electrolyte and enhancing the rate of lithium-ion transport inside the battery. It is worth noting that due to the strong intermolecular van der Waals forces and the nano-effect, Ni-HAB tends to agglomerate, making it difficult to form a uniform coating on the separator surface and resulting in a larger contact angle.

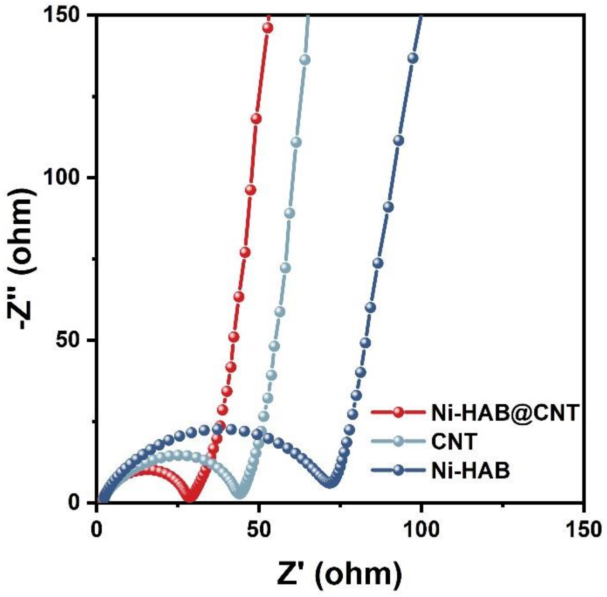

Figure S28. Nyquist plots of Li-S cells with Ni-HAB@CNT, CNT, and Ni-HAB modified separators before cycling.

EIS profiles were obtained for cells equipped with various separators before cycling. All three cells displayed a typical semicircle in the EIS spectra, corresponding to the ion transportation resistance (R_{ct}) in the high-frequency region. The R_{ct} of the cell with Ni-HAB@CNT modified separator was significantly lower than that of the other separators, corresponding to the strong electrolyte affinity and high conductivity of Ni-HAB@CNT.

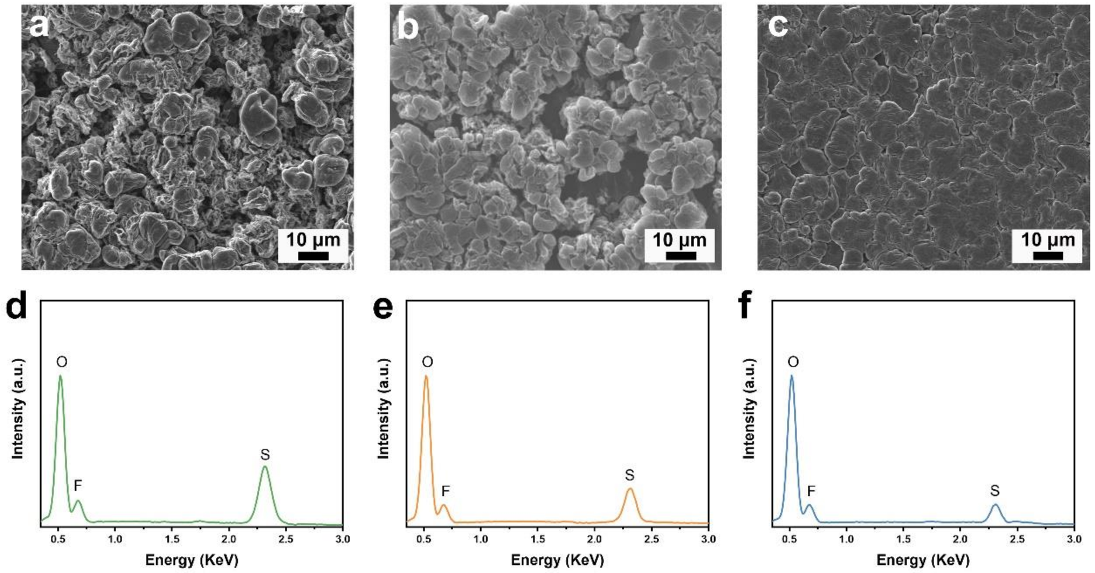

Figure S29. Post-mortem analyses of cycled Li-S battery anodes after cycles: SEM images of the cycled Li anodes that were assembled with (a) Ni-HAB/PP, (b) CNT/PP, and (c) Ni-HAB@CNT/PP separator, respectively. Corresponding EDS plots of the cycled Li anodes: (d) Ni-HAB, (e) CNT, and (f) Ni-HAB@CNT.

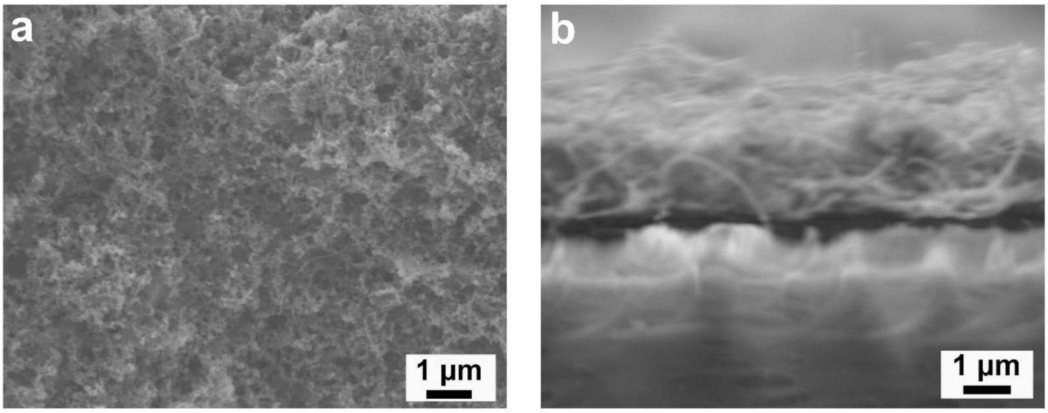

Figure S30. SEM images of (a) cathode and (b) separator cross-sections of the cell with Ni-HAB@CNT modified PP.

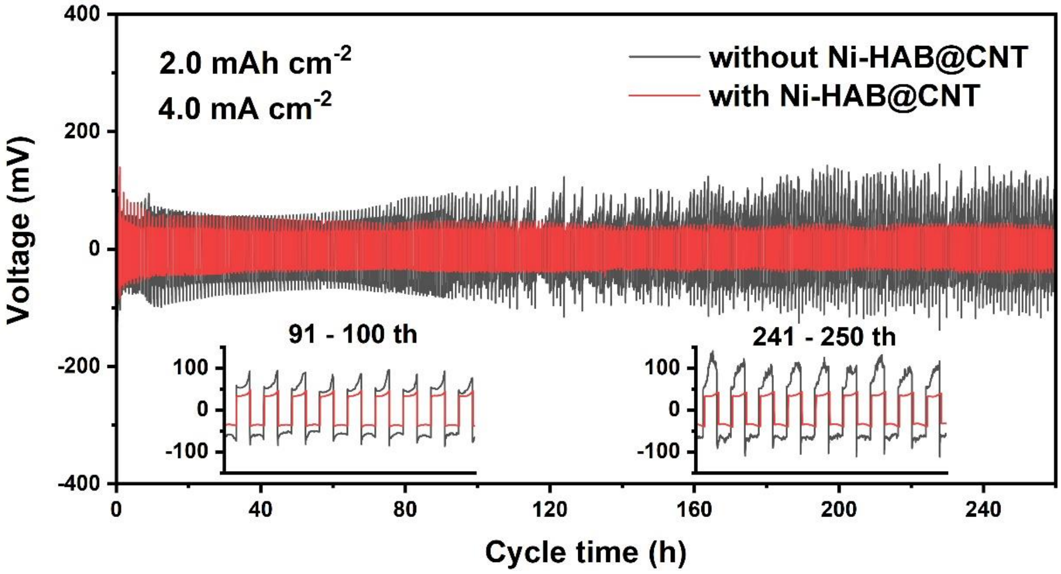

Figure S31. Cycle stability of Li || Li symmetric cells based on bare PP and Ni-HAB@CNT modified PP at 4.0 mA cm^{-2} and stationary capacity of 2 mAh cm^{-2}.

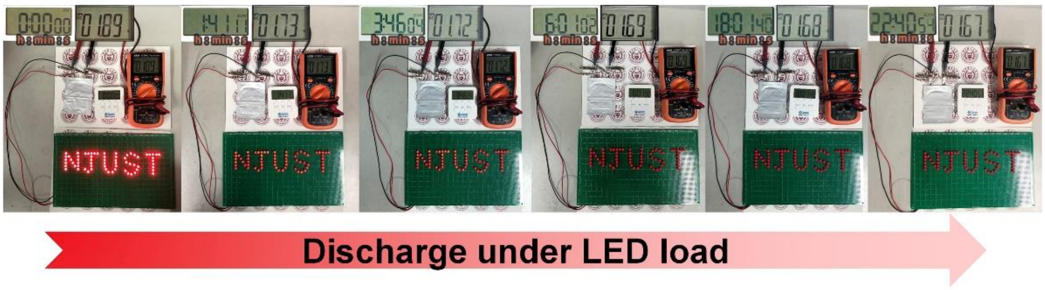

Figure S32. Digital photos of LED lights at different moments powered by the pouch cell with Ni-HAB@CNT modified PP separator.

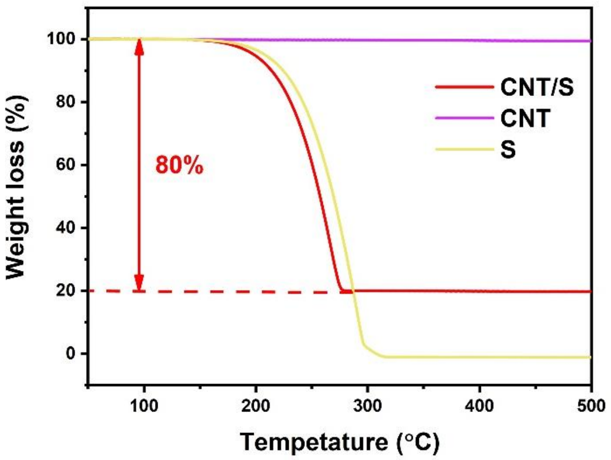

Figure S33. TGA curves of the S powder, CNT, and CNT/S compounds.

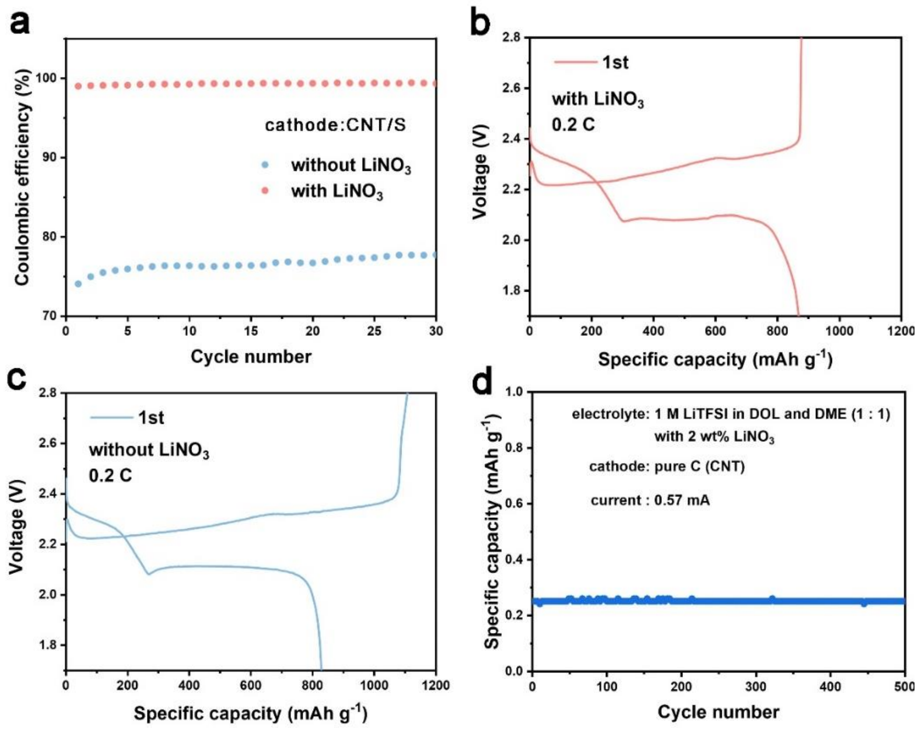

Figure S34. (a) Cycling coulombic efficiency of Li-S cells with different electrolytes; (b, c) Voltage profiles for the Li-S cells with different electrolytes; (d) Cycle performance of cells with super P cathode.

Electrolytes containing $LiNO_3$ additive are commonly used in non-anodic research on lithium-sulfur batteries.[13-14] In Li-S batteries, the main function of $LiNO_3$ is to build a solid electrolyte interface (SEI) film on the surface of lithium electrode, which can prevent the reaction between LPSs and lithium, thereby enhancing the coulombic efficiency of the battery.[15-16] As shown in Figure 33a-c, using the same CNT/S cathode, the electrolyte containing $LiNO_3$ can enhance the coulombic efficiency of the battery from below 80% to over 98%. Furthermore, during the first discharge cycle, the capacity of the battery using $LiNO_3$-containing electrolyte only slightly increases compared to the battery without $LiNO_3$. This may be due to the higher concentration of lithium ions in the electrolyte resulting from the introduction of $LiNO_3$. Subsequent experiments with sulfur-free batteries revealed that $LiNO_3$

provides almost negligible capacity contribution within the working voltage range of LSBs (1.7 – 2.8 V, Figure S34d).

Table S1. Physical properties of the prepared samples

Sample	CNT	Ni-HAB	Ni-HAB@CNT
BET Surface areas ($m^2\ g^{-1}$)	197.9063	107.6791	121.0759
Total pore volume ($cm^3\ g^{-1}$)	0.567922	0.191254	0.234472

Table S2. Comparison between the Ni-HAB@CNT modified PP and other MOF-based modified separators.

Sample	Cycling performance [mAh g^{-1}]	Decay rate (per cycle, %)	Rate performance [mAh g^{-1}]	Ref
Mn-BTC	1100 (80th, 0.1C)	0.28	100 (5C)	S17
$Ni_3(HITP)_2$	1139 (100 th, 0.2C)	0.2	589 (5C)	S18
CNT@ZIF-8	870.3 (100th, 0.2C)	0.45	583.2 (2C)	S19
UiO-66-S/Nafion	872.3 (200th, 0.1C)	0.11	785 (3C)	S20
B/2D MOF-Co	703 (200th, 0.5C)	0.14	478 (5C)	S21
HKUST-1@GO	799 (500th, 0.5C)	0.06	488 (3C)	S22
MIL-125 (Ti)	726 (200th, 0.2C)	0.2	592 (2C)	S23
Ni-HAB@CNT	1070 (200th, 0.2C)	0.07	799 (3C)	This work

Table S3. Comparison of specifications for different materials as PP separator modification layers

Sample	Thickness (μm)	Mass (mg cm^{-2})	Ref
FeSC@NSC	30	1.0	S24
Fe-NSxC	15	0.42 - 0.45	S25
VN@NC	3.3	0.25	S26
CNT-OH+TDI+THPP	17	-	S27
RHCF	10 – 50	-	S28
$Se_{0.06}$SPAN/MMT	10	0.5	S29
C-Lepidolite	19.2	0.995	S30
CoP/C	17.7	0.3	S31
Co-Nx@NPC	41.3	-	S32
ZnS-SnS@NC	10	0.5	S33
Ni-HAB@CNT	1.1	0.15 mg cm^{-2}	This work

References

[1] D. Moy, A. Manivannan, S. R. Narayanan, *J. Electrochem. Soc.* **2014**, 162, A1.

[2] F. Y. Fan, W. C. Carter, Y.-M. Chiang, *Adv. Mater.* **2015**, 27, 5203.

[3] G. Kresse, D. Joubert, *Phys. Rev. B* **1999**, 59, 1758.

[4] G. Kresse, J. Furthmüller, *Phys. Rev. B* **1996**, 54, 11169.

[5] J. P. Perdew, K. Burke, Y. Wang, *Phys. Rev. B* **1996**, 54, 16533.

[6] M. Methfessel, A. T. Paxton, *Phys. Rev. B* **1989**, 40, 3616.

[7] H. J. Monkhorst, J. D. Pack, *Phys. Rev. B* **1976**, 13, 5188.

[8] G. Henkelman, B. P. Uberuaga, H. Jónsson, *J. Chem. Phys.* **2000**, 113, 9901.

[9] G. Henkelman, A. Arnaldsson, H. Jónsson, *Comput. Mater. Sci.* **2006**, 36, 354.

[10]

[11] Z. Du, X. Chen, W. Hu, C. Chuang, S. Xie, A. Hu, W. Yan, X. Kong, X. Wu, H. Ji, L.-J. Wan, *J. Am. Chem. Soc.* **2019**, 141, 3977.

[12] G. Zhou, S. Zhao, T. Wang, S.-Z. Yang, B. Johannessen, H. Chen, C. Liu, Y. Ye, Y. Wu, Y. Peng, C. Liu, S. P. Jiang, Q. Zhang, Y. Cui, *Nano Lett.* **2020**, 20, 1252.

[13] Z. Li, I. Sami, J. Yang, J. Li, R. V. Kumar, M. Chhowalla, *Nat. Energy* **2023**, 8, 84.

[14] X. Sun, Y. Qiu, B. Jiang, Z. Chen, C. Zhao, H. Zhou, L. Yang, L. Fan, Y. Zhang, N. Zhang, *Nat. Commun.* **2023**, 14, 291.

[15] X. Liang, Z. Wen, Y. Liu, M. Wu, J. Jin, H. Zhang, X. Wu, *J. Power Sources* **2011**, 196, 9839.

[16] S. Xiong, K. Xie, Y. Diao, X. Hong, *Electrochim. Acta* **2012**, 83, 78.

[17] S. Suriyakumar, M. Kanagaraj, M. Kathiresan, N. Angulakshmi, S. Thomas, A. M. Stephan, *Electrochim. Acta* **2018**, 265, 151.

[18] Y. Zang, F. Pei, J. Huang, Z. Fu, G. Xu, X. Fang, *Adv. Energy Mater.* **2018**, 8, 1802052.

[19] F. Wu, S. Zhao, L. Chen, Y. Lu, Y. Su, Y. Jia, L. Bao, J. Wang, S. Chen, R. Chen, *Energy Stor. Mater.* **2018**, 14, 383.

[20] S. H. Kim, J. S. Yeon, R. Kim, K. M. Choi, H. S. Park, *J. Mater. Chem. A* **2018**, 6, 24971.

[21] Y. Li, S. Lin, D. Wang, T. Gao, J. Song, P. Zhou, Z. Xu, Z. Yang, N. Xiao, S. Guo, *Adv. Mater.* **2020**, 32, 1906722.

[22] S. Bai, X. Liu, K. Zhu, S. Wu, H. Zhou, *Nat. Energy* **2016**, 1, 16094.

[23] C. Qi, L. Xu, J. Wang, H. Li, C. Zhao, L. Wang, T. Liu, *ACS Sustain. Chem. Eng* **2020**, 8, 12968.

[24] X. Zuo, M. Zhen, D. Liu, H. Yu, X. Feng, W. Zhou, H. Wang, Y. Zhang, *Adv. Funct. Mater.* **2023**, 33, 2214206.

[25] L. Ren, J. Liu, Y. Zhao, Y. Wang, X. Lu, M. Zhou, G. Zhang, W. Liu, H. Xu, X. Sun, *Adv. Funct. Mater.* **2023**, 33, 2210509.

[26] F. Ma, Z. Chen, K. Srinivas, D. Liu, Z. Zhang, Y. Wu, M.-q. Zhu, Q. Wu, Y. Chen, *Chem. Eng. J.* **2023**, 459, 141526.

[27] B. Yang, D. Guo, P. Lin, L. Zhou, J. Li, G. Fang, J. Wang, H. Jin, X. a. Chen, S. Wang, *Angew. Chem.* **2022**, 134, e202204327.

[28] L. Huang, S. Shen, Y. Zhong, Y. Zhang, L. Zhang, X. Wang, X. Xia, X. Tong, J. Zhou, J. Tu, *Adv. Mater.* **2022**, 34, 2107415.

[29] W. Wang, K. Xi, B. Li, H. Li, S. Liu, J. Wang, H. Zhao, H. Li, A. M. Abdelkader, X. Gao, G. Li, *Adv. Energy Mater.* **2022**, 12, 2200160.

[30] G. Zeng, Y. Liu, D. Chen, C. Zhen, Y. Han, W. He, *Adv. Energy Mater.* **2021**, 11, 2102058.

[31] J. Lin, K. Zhang, Z. Zhu, R. Zhang, N. Li, C. Zhao, *ACS Appl. Mater. Interfaces* **2020**, 12, 2497-2504.

[32] Z. Cheng, H. Pan, J. Chen, X. Meng, R. Wang, *Adv. Energy Mater.* **2019**, 9, 1901609.

[33] W. Yao, W. Zheng, J. Xu, C. Tian, K. Han, W. Sun, S. Xiao, *ACS Nano* **2021**, 15, 7114–7130.
